# *Origanum majorana* L. Extract Attenuated Benign Prostatic Hyperplasia in Rat Model: Effect on Oxidative Stress, Apoptosis, and Proliferation

**DOI:** 10.3390/antiox11061149

**Published:** 2022-06-11

**Authors:** Dalia Mahmoud Abdelmonem Elsherbini, Hailah M. Almohaimeed, Mohamed El-Sherbiny, Zuhair M. Mohammedsaleh, Nehal M. Elsherbiny, Sami A. Gabr, Hasnaa Ali Ebrahim

**Affiliations:** 1 Department of Clinical Laboratory Sciences, College of Applied Medical Sciences, Jouf University, Sakaka 42421, Saudi Arabia; dmelsherbini@ju.edu.sa; 2 Department of Anatomy, Faculty of Medicine, Mansoura University, Mansoura 35516, Egypt; dr_hasnaa_ali@mans.edu.eg or; 3 Department of Basic Medical Sciences, College of Medicine, Princess Nourah Bint Abdulrahman University, Riyadh 11671, Saudi Arabia; hmalmohaimeed@pnu.edu.sa; 4 Department of Basic Medical Sciences, College of Medicine, AlMaarefa University, Riyadh 11597, Saudi Arabia; msharbini@mcst.edu.sa; 5 Department of Medical Laboratory Technology, Faculty of Applied Medical Sciences, University of Tabuk, Tabuk 71491, Saudi Arabia; zsaleh@ut.edu.sa; 6 Department of Pharmaceutical Chemistry, Faculty of Pharmacy, University of Tabuk, Tabuk 71491, Saudi Arabia; drnehal@mans.edu.eg; 7 Department of Biochemistry, Faculty of Pharmacy, Mansoura University, Mansoura 35516, Egypt

**Keywords:** BPH, *Origanum majorana*, finasteride, oxidative stress, proliferation, apoptosis

## Abstract

Benign prostatic hyperplasia (BPH) is a widespread androgenic illness influencing elderly men. It is distinguished by prostatic epithelial and stromal muscle cell proliferation. Inflammation, oxidative stress, and apoptosis have all been interrelated to the development of BPH. Marjoram (*Origanum majorana* L.) is a herb with reported antiproliferative, proapoptotic, and antioxidative properties, which have not yet been studied in relation to BPH. Consequently, in this work, an ethanolic extract of *O. majorana* was prepared in two doses (250 and 500 mg/kg/day) to be injected into castrated rats after induction of a testosterone-BPH model. Testosterone propionate (TP) was subcutaneously injected (0.5 mg/kg/day) for one week after castration to induce BPH. Forty adult Wistar male rats were randomly allocated into five groups: control, BPH model, high and low *O. majorana* doses (250, 500 mg/kg/day), and finasteride (FN) (0.8 mg/kg/day) as a positive control. Treatment was continued with drugs/normal saline for 28 days. Rat’s body and prostate were weighed, prostate index (PI) and % of prostate growth inhibition were calculated, serum dihydrotestosterone (DHT), prostatic content of superoxide dismutase (SOD), catalase (CAT), total antioxidant capacity (TAC), and malondialdehyde (MDA), DN damage, histopathological changes, immune expression of proliferating cell nuclear antigen (PCNA), caspase-3, α-SMA, and TGF-β1 were assessed. In addition, molecular quantitative PCR and ELISA analyses were performed to identify the expression of mRNAs and related proteins of both caspase-3 and TGF-β1 in prostate tissue from *O. majorana*-treated and untreated groups. Rats with BPH had significantly higher prostate weights and PI, higher DHT, DNA damage (8-hydroxyguanine, 8-OH-dG), and MDA levels with prominent PCNA, α-SMA, and TGF-β expression, but lower SOD, CAT, and TAC activity and caspase-3 expression. *O. majorana* (250 and 500 mg/kg/day)-treated groups revealed a decrease in prostate weights and PI, lower levels of DHT, suppressed oxidative stress, reduced tissue proliferation and fibrosis, and restored antioxidant and proapoptotic activity. Additionally, quantitative PCR and ELISA analysis showed that treatment with *O. majorana* significantly upregulated the expression of caspase-3 and downregulated the expression of TGF-β in prostate tissues of BPH rats. The data were confirmed by the immunohistological reactivity of these targeted markers in the prostate tissues. These effects were more significant with *O. majorana* 500 mg/mL/rat. In conclusion, the current study indicates the efficient use of *O. majorana* in the treatment of testosterone-induced BPH through its antiproliferative, proapoptotic, and antioxidative mechanisms.

## 1. Introduction

Benign prostatic hyperplasia (BPH) is a prevalent androgenic illness affecting elderly men. It is marked by epithelial and stromal muscle cell proliferation within the prostate, resulting in lower urinary tract manifestations [1,2]. Although numerous attempts have been made throughout the years, the pathophysiology of BPH has yet to be fully understood. BPH is caused by a combination of factors, including testicular hormones and age. Furthermore, the involvement of proliferation/apoptosis and oxidation/antioxidation pathways was documented in the progression of BPH and validated by several studies over the years [3,4,5]. For most patients, medication is still the primary line of treatment. Alpha-blockers and 5α-reductase inhibitors are the two routinely prescribed medications for BPH. Alpha-blockers, such as alfuzosin, doxazosin, tamsulosin, and terazosin, relax the prostate’s smooth muscle fibers, reducing the dynamic component of prostatic obstruction [6]. The use of α-reductase inhibitors (e.g., finasteride) as a medical therapy for BPH prevents testosterone conversion to dihydrotestosterone (DHT), lowers DHT levels, and suppresses hyperplastic growth of the prostate [7] with a 20–30% reduction in the prostate size [8,9]. However, finasteride-related side effects are frequently documented, such as gynecomastia, headaches, vertigo, chest infections and distress, decreased libido, erectile disturbance, and male infertility owing to low sperm production [10,11]. Such adverse effects limit the use of standard BPH medication and necessitate further research for alternative safe treatment.

Phytotherapeutics have recently received much attention as an efficient and safe treatment strategy for BPH patients, and many plants have shown anti-BPH effects [12]. Several traditional medicine prescriptions or herbs have been reported for their successful impact on BPH, such as Saw palmetto and *Rubus coreanus* [13], Zi-Shen Pill (ZSP), which consists of different medicinal plants [14], *Curcuma longa* [15], *Scutellaria baicalensis* [16], Yukmijihwang-tang [17], curcumin [18] and *Cinnamomi cortex* [19]. Marjoram (*Origanum majorana* L.) is a herb with fragrant leaves that is commonly used in flavoring and culinary applications [20,21]. Interestingly, diverse experimental models have proved their efficiency in many of the folklore therapeutic claims [20]. Moreover, it is a popular home cure for respiratory tract infections, cough, rheumatoid arthritis, cardiovascular illness, neurological disorders, epilepsy, sleeplessness, skin care, flatulence, and stomach illnesses [21,22,23]. Regarding chemical composition, *O. majorana* extract contains phenolic terpenoids (carvacrol, thymol), flavonoids (luteolin, diosmetin, apigenin), hydroquinone, tannins, phenolic glycosides (vitexin, thymonin, arbutin, methyl arbutin, orientin) and sitosterol [24]. *O. majorana* was considered to be generally safe by the Food and Drug Administration. In this context, alcoholic and aqueous extracts of *Origanum majorana* showed no toxic effects on the liver, kidney, and chromosomes when administrated to mice [25]. In addition, Seoudi et al. [26] reported no mortalities in rats following oral administration of *O. majorana* at doses up to 5 g/kg of body weight.

Numerous studies linked its apparent antioxidant activity to the elevated phenolic content [23]. Additionally, previous research work reported antiproliferative efficacy and robust proapoptotic benefits of *O. majorana* in various types of malignancies [24]. In this context, many studies found that *O. majorana* ameliorated human cancer cell lines’ growth, including human lymphoblastic leukemia cell lines, human hepatoma cell lines, in addition to experimentally induced fibrosarcoma, lung carcinoma, and hormone-dependent prostate carcinoma [24,27,28]. Previous reports also revealed the potent antiproliferative impact of *O. majorana* on colon cancer cells through triggering apoptosis with mitochondrial dysfunction [29,30,31].

The overall goal of this study is to investigate the mitigating effect of *O. majorana* extract on experimentally induced BPH. The antiproliferative, apoptotic, and antioxidative properties of *O. majorana* were compared to a standard medication, finasteride, using immunohistochemistry, ELISA, quantitative PCR, and biochemical measurements.

## 2. Materials and Methods

The G*Power program for Windows (version 3.1.9.7) was used to compute the sample size according to the procedures specified [32]. Following previous studies [5,33,34,35,36,37], we assumed that the means for five groups (vehicle, BPH, finasteride, *O. majorana* 250 and 500 mg/mL) would be (5.50, 10.17, 7.50, 23.82, and 5.63) for MDA, and (800.05, 567.06, 658.23, 115.20 and 991.462) for SOD. Considering the standard deviation (SD) within each group, 1.24 for MDA and 105.168 for SOD, effect size (f) would be 4.67 for MDA and 2.635 for SOD. According to these assumptions, sample sizes of MDA and SOD of 10 and 10, respectively, achieve 95% power to identify these effect sizes at an alpha level of 5%, with sample sizes of two per group in a one-way ANOVA design with five groups. Using the F-Test with a significance level of 0.05, the total sample of 10 achieves a power of 95%. For morphometric studies, we assumed eight rats per group.

### 2.1. Materials

*O. majorana* belongs to the family Lamiaceae and is popularly known as sweet marjoram. It was obtained as a whole plant from a marketplace in Egypt. To prepare an ethanolic extract of *O. majorana*, 25 g of *O. majorana* flowers were immersed in 150 mL of ethanol for 24 h before being re-immersed for another 24 h (2× extraction times). A rotary evaporator was used to concentrate the extracts and then dried in a lyophilizer (LABCONCO lyophilizer, shell, freeze system, Kansas city, MO, USA) at 37–40 °C, then kept in a desiccator until needed [38]. In distilled water, the dried extract was dissolved, and a final stock with a concentration of 250 and 500 mg/kg/day was prepared for further usage. The selection of doses was based on preliminary experiments and previous studies [36,38].

Finasteride and testosterone propionate (TP) were purchased from Sigma Aldrich; Merck KGaA (Darmstadt, Germany). SOD, CAT, MDA, DNA damage (8-hydroxyguanine, 8-OH-dG), total antioxidant capacity (TAC), testosterone, and DHT assay kits were purchased from Abcam (Cambridge, MA, USA).

#### Animals

Forty adult Wistar male rats aged 6–8 months, weighing 250–300 g, were used. Rats were housed in a clean facility at 25 °C with a relative humidity of 55% and under normal laboratory settings with a regular 12 h light/dark cycle. Rats were provided unrestricted access to normal laboratory feed and water. The rats, after one week of acclimatization, were assigned to experimental groups at random.

### 2.2. Methods

#### 2.2.1. Testosterone Propionate-Induced Rat Model of BPH

**Figure 1:**
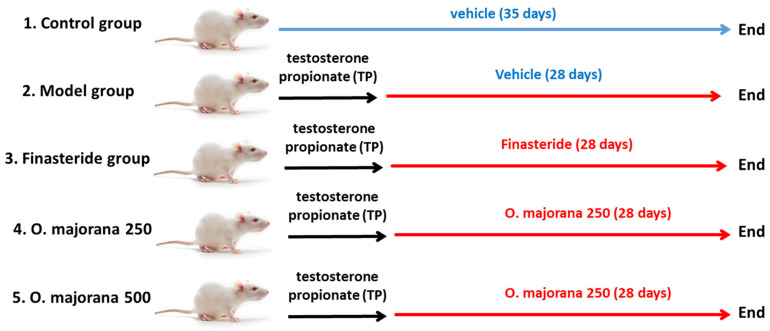
Flow chart of animal groups.

Rats were allocated into 5 groups (*n* = 8) at random (Figure 1): control group (control/vehicle), non-treated rats injected intraperitoneally (i.p.) with the vehicle, while the other four groups were subjected to a well-sterile castration procedure to extract both testes after being anesthetized with phenobarbital injection (i.p., 50 mg/kg) to exclude the effect of intrinsic testosterone. All castrated rats received subcutaneous (S.C.) testosterone propionate (TP) (dissolved in corn oil) at 0.5 mg/kg daily following castration for one week. The BPH model was established, and the therapy was administrated within the 28 consecutive days of TP injection [39]. One group was assigned as the model group, and the other three were designated as treated groups; the Finasteride group (FN), in which rats received intragastric dissolved finasteride in ultrapure water (0.8 mg/kg/day) and were considered a positive control group; marjoram low dose group (*O. majorana* 250), rats were treated with intragastric (i.g.) marjoram extract (250 mg/kg/day) for 28 days; marjoram high dose group (*O. majorana* 500 mg/kg/day), rats were treated with intragastric (i.g.) of marjoram extract for 28 days. At the end of the experiment (29th day), blood samples (~6–8 mL) were withdrawn from rats under anesthesia (intravenous injection of 3% pentobarbital sodium at 39 mg/kg body weight, Sigma-Aldrich; Merck KGaA, Darmstadt, Germany, cat no. P3761). Rats were euthanized, and the prostate was excised through a median abdominal incision after exposing the bladder. Ventral (VP) and lateral (LP) lobes of harvested prostates were dissected based on their position relative to the urinary bladder. One-half of each lobe was fixed for 24 h in neutral 10% formalin at room temperature for histological and immunohistochemical processing, while for subsequent biochemical analysis, the remaining half was kept at −80 °C.

To test the safety of *O. majorana* extract, a group of normal rats received *O. majorana* 500 mg/kg/day for 35 days. Thereafter, animals were placed in metabolic cages to collect urine for assessment of protein (Spinreact, Barcelona, Spain), and blood was collected and centrifuged to separate the serum for assessment of kidney and liver function. Serum creatinine (Diamond Diagnostic Chemical Company, Budapest, Hungary), blood urea nitrogen (BUN, BIOMED Diagnostic, Cairo, Egypt), alanine aminotransferase (ALT, MAK052), and aspartate aminotransferase (AST, MAK055) were evaluated according to manufacturers’ instructions. Additionally, sections from the liver and kidneys were harvested and processed for the histopathological study of possible tissue alterations using Hematoxylin and Eosin staining. Results are shown as Supplementary Materials.

#### 2.2.2. Body Weight and Prostate Weight

Rats were weighed at the start of the experiment, weekly, and at the end. Excised prostate from each rat was weighed to calculate the prostate index following the formula that was previously described by Akbari et al. [40].

$$\mathrm{Prostate}\;\mathrm{index}=\frac{\mathrm{Prostate}\;\mathrm{weight}}{\mathrm{Body}\;\mathrm{weight}}$$

Increased prostate weight is a significant marker of BPH as it reflects prostatic hyperplasia (epithelial and stromal), which is linked to prostate weight [17]. Changes in the prostate index upon comparing each group with vehicle and model groups distinguish the percentage of inhibition of disease progression with treatment [40].

$$\mathrm{Percentage}\;\mathrm{of}\;\mathrm{inhibition}=100\;-\;\left[\frac{\mathrm{Treatment}\;\mathrm{group}\;-\;\mathrm{Control}\;\left(\mathrm{Vehicle}\right)}{\mathrm{Model}\;\mathrm{group}\;-\;\mathrm{Control}\;\left(\mathrm{Vehicle}\right)}\times 100\right]$$

#### 2.2.3. Hormonal Assessment

Collected blood samples were centrifuged for 15 min (2000× *g*, 4 °C) to extract serum, which was then stored at −80 °C. Serum testosterone and DHT levels were determined using an enzyme-linked immunosorbent assay (ELISA) according to the manufacturer’s protocol. Sample absorbance was measured at 450 nm using an Epoch Micro plate Spectrophotometer (BioTek Instruments, Inc., Winooski, VT, USA). The recorded values were in ng/mL [41].

#### 2.2.4. Measurement of Oxidant-Antioxidant Status in Prostate Tissues

Homogenization of prostate tissues was performed using ice-cold phosphate-buffered saline (50 mM potassium phosphate pH 7.5). Cellular oxidant-antioxidant parameters, MDA, DNA damage (8-hydroxyguanine, 8-OH-dG), SOD and CAT activities, and cellular total antioxidant capacity (TAC) were identified in prostate tissues of control, *O. majorana* PBH-treated, and non-treated groups. Prostate MDA, lipid peroxidation marker, concentration was detected by spectrophotometer according to the manufacturer’s instructions, following the Satoh protocol [42]. The absorbance was determined at 532 nm, and butanol was used as a blank. MDA measurements were presented in nmol/g protein. 8-Hydroxyguanine (8-OH-dG) was measured as a DNA damage marker using Elisa kits (Abcam; Cambridge, MA, USA), and the results were obtained in nanograms per milliliter as previously reported [43]. In addition, the activity of both SOD and CAT antioxidant enzymes, as well as cellular TAC, were measured in prostate tissues as previously analyzed [33,44,45,46].

#### 2.2.5. Assessment of Nrf2 and HO-1 in Prostate Tissues by ELISA

Nrf2 and HO-1 levels were quantified in both *O. majorana*-treated and non-treated prostate tissues by immunoassay analysis using an ELISA kit (cat. no. SU-B30429; Shanghai Yuchun Biotechnology, Shanghai, China) for Nrf2 and ELISA kit (cat. no: E-EL-R0488, Elabscience, Houston, TX 77079, USA) for the determination of HO-1, respectively. In this test, 50 mg of the tissue sample was homogenized using PBS buffer with PMSF as a serine inhibitor. Then, the homogenized tissue samples were centrifuged at 15,000 rpm, followed by the collection of the supernatant. To determine the level of the respective Nrf2 and HO-1 concentrations, the produced supernatant was added to each well of the ELISA plate and incubated with respective antibodies for Nrf2 and HO-1 for 60 min. Finally, the levels of the resultant Nrf2 and HO-1 concentrations were determined using an ELISA reader at 450 nm. Data were interpolated from the standard curves to calculate the amount of Nrf2 (pg/mg) and HO-1 (pg/mg), respectively.

#### 2.2.6. Assessment of Caspase-3 and TGF-β1 in Prostate Tissues by ELISA

Both caspase-3 and TGF-β1 were identified in prostate tissues using commercially available ELISA kits (Millipore Corporation, Billerica, Santa Clara, MA, USA) for caspase-3 and (Aviscera Bioscience, CA, USA) for TGF-β1 and the results were obtained in nanograms per milliliter [43,47]. Each test was performed in triplicate.

#### 2.2.7. Quantitative Real-Time PCR Analysis for Caspase-3 and TGF-β1 in Prostate Tissues

(a) Isolation of total RNA and complementary DNA (cDNA) Synthesis

According to the manufacturer’s instructions, TRIzol reagent (Invitrogen, Bedford, MA, USA) was used to isolate the total RNA of cells ^46^. Then, reverse transcription with Oligo (dT) and M-MLV Reverse Transcriptase (Thermo Fisher Scientific, Bedford, MA, USA) was applied to obtain a purified RNA sample. Finally, real-time polymerase chain reaction (PCR) was applied by an ABI 7900HT system using SYBR^®^ Premix (Takara, Dalian, China) to synthesize complementary DNA (cDNA) from purified RNA samples as mentioned previously in the literature. The procedures were performed according to the manufacturer’s instructions.

(b) The real-time PCR analysis process

The expression of targeted mRNAs for caspase-3 and TGF-β1 in prostate tissues were identified by corresponding primers as shown in Table 1 in the thermal cycler process at different steps of heat conditions as follows: denaturation at 9 °C for 10 s, 45 cycles at 95 °C for 10 s, and 65 °C for 30 s [46]. The PCR products obtained were matched with glyceraldehyde-3-phosphate dehydrogenase (GAPDH) as the reference gene [48]. No nonspecific amplification was observed. The data were analyzed using the comparison Ct (2^−ΔΔCt^) method and were expressed as fold changes relative to the respective controls. Each sample was analyzed in triplicate [48].

#### 2.2.8. Histopathological Examination

For the histological examination, 4 μm thick sections were prepared from prostate paraffin blocks after fixing the prostate tissue in 4% paraformaldehyde (Sigma-Aldrich). The sections were stained with (H&E) and Masson’s trichrome stain [49] and were photographed using a light microscope (BX-51; Olympus, Tokyo, Japan).

#### 2.2.9. Immunohistochemical Staining Technique

Deparaffinized 4 μm sections were placed on coated slides and then treated for ten minutes at room temperature with hydrogen peroxide (0.3%/methanol) to stop the activity of endogenous peroxidase. Finally, sections were heated for ten minutes at 95 °C in 10 mM citrate buffer to induce antigen retrieval before being allowed to cool for 60 min.

Sections were incubated with the following primary antibodies for immunostaining: gamma H2Ax and Poly (ADP-ribose) polymerase 1 (PARP1), proliferating cell nuclear antigen (PCNA) [50], caspase-3, TGF-β1 [51], and alpha smooth muscle actin (α-SMA) [52] as presented in Table 2. Then, they were left in phosphate-buffered saline (PBS) at 4 °C overnight before adding the universal secondary anti-mouse IgGκ (sc-516102, Santa Cruz, CA, USA) or anti-rabbit IgG (sc-2357, Santa Cruz) for 30 min to localize the primary antibody binding followed by washing again with PBS. Finally, Diaminobenzidine (DAB) was added for four minutes to demonstrate peroxidase activity, followed by hematoxylin staining as counterstain [53]. Olympus^®^ CX41 light microscope was used for examining the sections that have been photographed by its digital camera Olympus^®^ SC100.

#### 2.2.10. Morphometric Analysis

ImageJ^®^ software win64 (Wayne Rasband NIH, Bethesda, MA, USA) [54] was applied to conduct morphometric measurements, including at least 5 sections per each prostatic lobe (ventral and lateral), and from each section, 5 different non-overlapping fields were examined (25 fields/prostatic lobe). Measurements were performed by one investigator blind to the work to avoid inter-observer errors. The following parameters were assessed:

(1) In H&E-stained sections, the prostatic epithelial height (μm) was measured by manually drawing a line through the acinar epithelia at a magnification of ×400. The acinar luminal area (μm^2^) was measured by drawing a line around the luminar perimeter and calculating the acinar area at a magnification of ×100
(2) Mean area % of collagen fiber was determined in the Masson’s trichrome-stained sections at a magnification of ×100
(3) Mean area % of positive immunoreactivity for (α-SMA) and (TGF-*B*1) was measured at a magnification of ×400.

Proliferative and apoptotic indices: positive immunoreactive cells for PCNA and caspase-3 were detected using the QuPath program (0.1.2) [55] in the tissue section at a magnification of X100. This method was applied to serial sections stained with PCNA and caspase-3 with five random fields of 1.2 mm^2^ analyzed on each section. For immunohistochemistry quantitative assessment of γH2Ax and PARP1, the Allred score was used [56,57], presented with a scale of 0–8, and quantified using the QuPath program (0.1.2).

#### 2.2.11. Statistical Analysis

Graphpad Prism, version 8, was used to analyze the data in this research. The independent samples *t*-test was used to compare the means of the two groups. A one-way analysis of variance (ANOVA) and Tukey’s post-hoc test were performed for multiple groups. The *p*-values for significance were set at *p* ˂ 0.05. The data were provided as a mean ± standard deviation (SD).

## 3. Results

### 3.1. Effect of O. majorana on the Rat’s Body Weight Gain and Prostate Weight

The BPH and *O. majorana* 250 groups gained less body weight than the finasteride and *O. majorana* 500 groups, which gained significantly more body weight than the control (vehicle) group. Considering that prostate weight is an essential indicator of BPH and that it is extremely difficult to obtain the initial prostate weight of rats during the experiment period, comparisons were made at the end of the study based on the weighed excised prostate in relation to the control (vehicle) group. When compared to the control/vehicle group, the BPH group had a significant increase in prostate weight (912.50 ± 85.39 mg) (Table 3). When compared to the BPH group, the finasteride and *O. majorana* 500 groups had a significant decrease in prostate weight (600.02 ± 81.64 and 637.50 ± 47.87 mg, respectively, *p* ˂ 0.05). The *O. majorana* 250 group had an insignificant reduction in prostate weight (812.50 ± 49.30 mg) relative to the BPH group. PI was significantly higher in the BPH group when compared with control or treated groups. The Finasteride group exhibited an evident decrease in PI, followed by *O. majorana 500* groups and *O. majorana* 250 groups.

PI inhibition percentages with finasteride, *O. majorana* high dose (500), and *O. majorana* low dose (250) were 58.75%, 51.70%, and 18.80%, respectively, while PI inhibition percentages relative to the control group were 52.29%, 47.43%, and 15.03%, respectively (Table 3).

### 3.2. Effect of O. majorana on DHT and Testosterone Serum Levels

**Figure 2:**
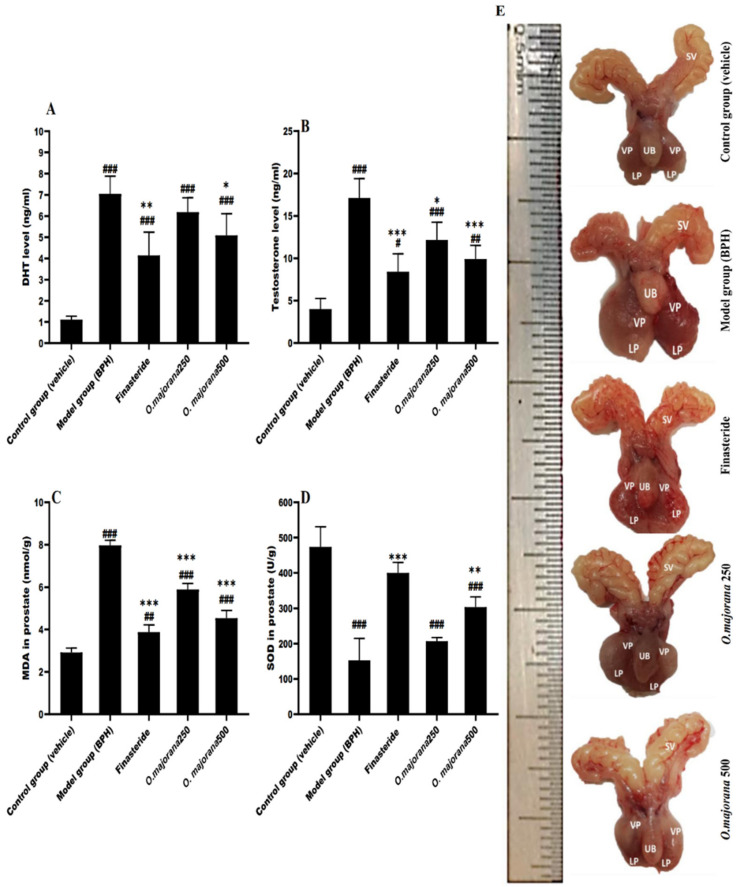
(**A**–**E**): Effect of *O. majorana* on serum hormone levels, MDA and SOD concentrations in prostatic tissue and morphological changes: (**A**) DHT serum level. (**B**) Testosterone serum level. (**C**) MDA concentration in prostatic tissue. (**D**) SOD concentration in prostatic tissue. Data are expressed as means ± SD (*n* = 8). ^###^ *p* < 0.001, ^##^ *p* < 0.01 and ^#^ *p* < 0.05 vs. control, *** *p* < 0.001, ** *p* < 0.01 and * *p* < 0.05 vs. BPH (model) group. (**E**) displays the variations in size of the prostate among the five groups: seminal vesicle (SV), urinary bladder (UB), ventral lobe (VP), and lateral lobe (LP).

DHT and testosterone are involved in expediting prostate hyperplasia and show a vital role in prostatic growth. The primary prostatic androgen is DHT. Serum DHT and testosterone levels are depicted in Figure 2. When compared to the control/vehicle group, the model (BPH) group had a significant increase in ±rum DHT levels (7.05 ± 0.83 ng/mL versus 1.11 ± 0.17 ng/mL, *p* < 0.001, Figure 2A). A significantly lower serum DHT levels were observed in the finasteride-treated group (4.14 ± 1.09 ng/mL, *p* < 0.01) relative to the BPH group. Similarly, DHT levels were less in the *O. majorana*-treated groups (6.18 ± 0.68 ng/mL in *O. majorana* 250 group, *p =* 0.05, 5.09 ± 0.99 ng/mL in the *O. majorana* 500 group, (*p* < 0.05)) compared to the BPH group. The difference between the *O. majorana* 500 and the finasteride-treated groups was insignificant (*p* > 0.05).

The BPH group had significant higher testosterone levels (17.14 ± 2.26 ng/mL) relative to the control/vehicle group (4.02 ± 1.24 ng/mL; *p* < 0.001; Figure 2B), whereas the finasteride-treated group (8.40 ± 2.12 ng/mL) had significantly lower testosterone levels than the BPH group (*p* < 0.001). Moreover, a marked decrease in testosterone levels was detected in the *O. majorana*-treated groups (12.16 ± 2.11 ng/mL in the *O. majorana* 250 group, *p* < 0.05; 9.90 ± 1.63 ng/mL in the *O. majorana* 500 group, *p* < 0.001) compared to the BPH group. The difference between *O. majorana* 250 and *O. majorana* 500 groups relative to the finasteride-treated group was insignificant (*p* > 0.05).

### 3.3. Effect of O. majorana on Prostatic MDA and SOD Levels

When compared to the control/vehicle group, the BPH group had a significant increase in the prostate lipid peroxidation marker MDA (7.97 ± 0.24 nmol/g versus 2.91 ± 0.21 U/g, *p* < 0.001, Figure 2C), whereas the finasteride-treated group (3.88 ± 0.35 nmol/g) had significantly lower prostate MDA content than the BPH group (*p* < 0.001). When compared to the BPH group, the *O. majorana*-treated groups (5.89 ± 0.28 nmol/g in *O. majorana* 250 group, *p* < 0.001; 4.54 ± 0.36 nmol/g in the *O. majorana* 500 group, *p* < 0.001) had a significantly lower prostate MDA content. Furthermore, when compared to the *O. majorana* 500 group, the *O. majorana* 250 groups revealed a significant difference in prostate MDA content (*p* < 0.001), while the finasteride-treated group showed an irrelevant difference (*p* > 0.05).

In contrast, the BPH group had significantly lower activity in prostatic SOD relative to the control/vehicle group (152.2 ± 62.73 U/g versus 437.6 ± 57.51 U/g, *p* < 0.001; Figure 2D). The finasteride-treated group revealed significantly higher activity in prostatic SOD (400.3 ± 29.72 U/g, *p* < 0.001) relative to the BPH group. When compared to the model group, *O. majorana*-treated groups had higher prostatic SOD levels (206.8 ± 10.34 U/g in *O. majorana* 250 group, *p =* 0.05; 303.5 ± 29.10 U/g in the *O. majorana* 500 group, *p* < 0.01). Moreover, when compared to *O. majorana* 500 groups, the finasteride-treated group had a non-significant difference (*p* > 0.05), whereas *O. majorana* 250 groups exhibited a significant difference (*p* < 0.05).

### 3.4. Effect of O. majorana on Prostate DNA Damage, CAT, and TAC

**Figure 3:**
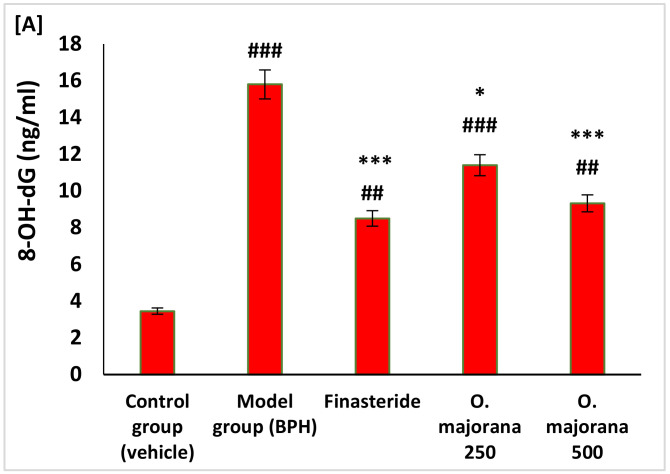

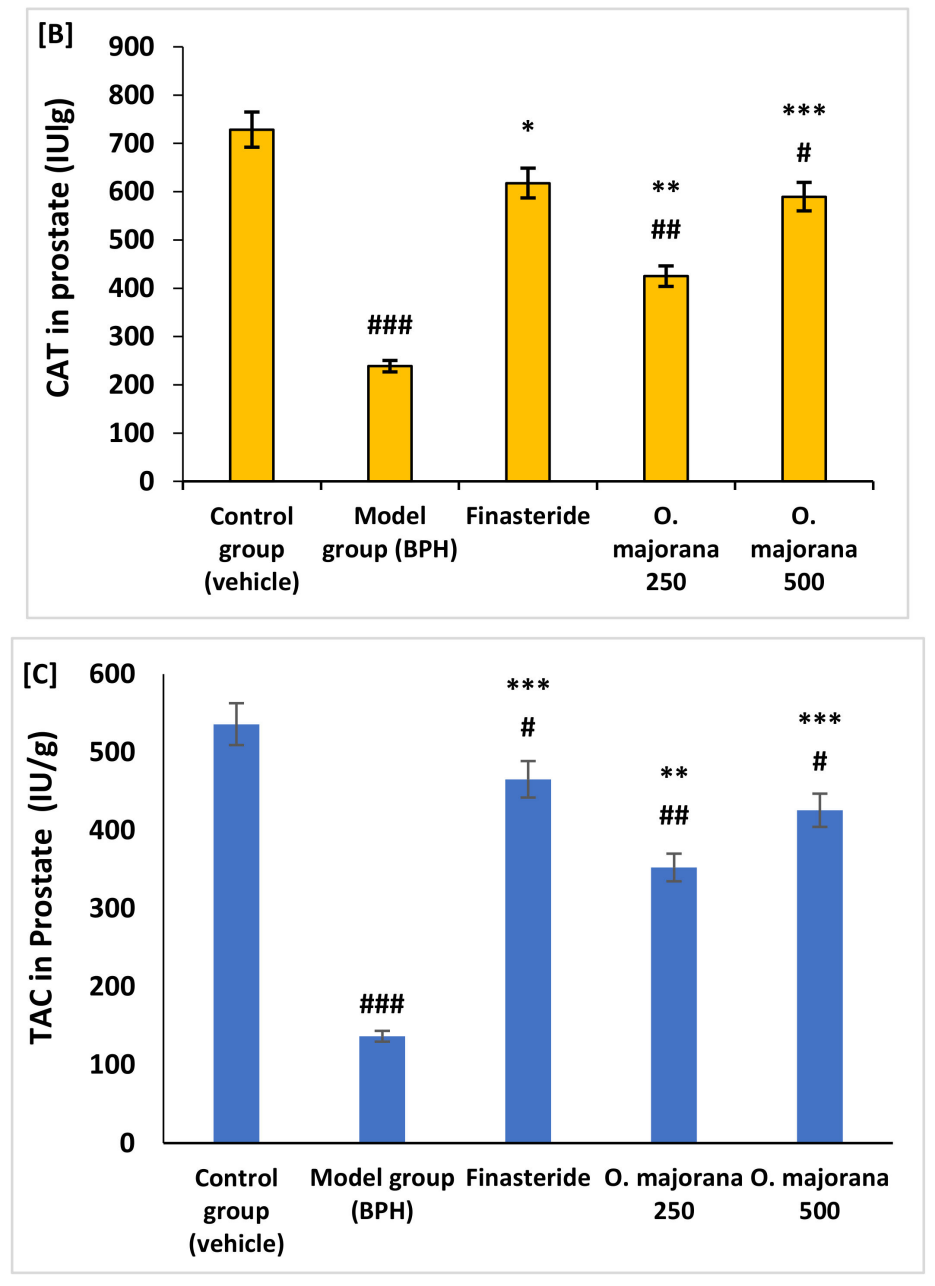
(**A**–**C**): Effect of *O. majorana* on cellular DNA damage (8-hydroxyguanine, 8-OH-dG), catalase (CAT) activity and total antioxidant capacity (TAC) in prostatic tissue: (**A**) cellular 8-OH-dG. (**B**) CAT tissue level. (**C**) TAC concentration in prostatic tissue. Data are expressed as means ± SD (*n* = 8). ^###^ *p* < 0.001, ^##^ *p* < 0.01 and ^#^ *p* < 0.05 vs. control, *** *p* < 0.001, ** *p* < 0.01 and * *p* < 0.01 vs. BPH (model) group.

In rats of the BPH (model) group, there was a significant increase in the levels of cellular DNA damage as measured by the levels of 8-hydroxyguanine (8-OH-dG) (*p* < 0.001) and a significant (*p* < 0.001) reduction in the expression levels of both CAT (Figure 2B), and TAC (Figure 2C), respectively, compared to control rats. Whereas *O. majorana*-treated BPH groups showed a significant reduction in the cellular DNA damage marker (8-OH-dG) and significantly increased cellular antioxidant status measured by CAT and TAC compared to BPH (model) (Figure 3A–C).

### 3.5. Effect of O. majorana on Nrf2 and HO-1 Levels in Prostate Tissue

**Figure 4:**
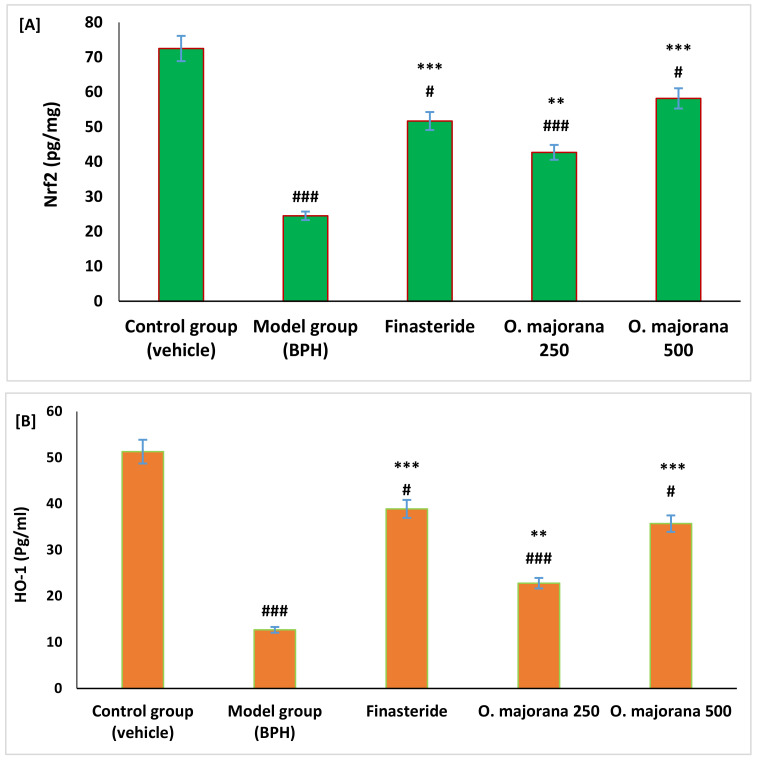
(**A**,**B**): Effect of *O. majorana* on protein of Nrf2 (**A**) and HO-1 (**B**) in prostatic tissue. Data are expressed as means ± SD (*n* = 8). ^###^ *p* < 0.001 and ^#^ *p* < 0.05 vs. control, *** *p* < 0.001 and ** *p* < 0.01 vs. BPH (model) group.

Results showed that Nrf2 and HO-1 proteins were significantly reduced in rats with BPH compared to control rats (*p* *<* 0.01). However, BPH rats treated with *O. majorana* at doses of 250 and 500 mg/kg/day showed a significant increase in levels of protein of both Nrf2 and HO-1 antioxidant parameters compared to rats of both BPH and the finasteride groups as measured by ELISA immunoassay techniques (Figure 4A,B).

### 3.6. Effect of O. majorana on mRNAs and Proteins of Caspase-3 and TGF-β1 in Prostate Tissue

**Figure 5:**
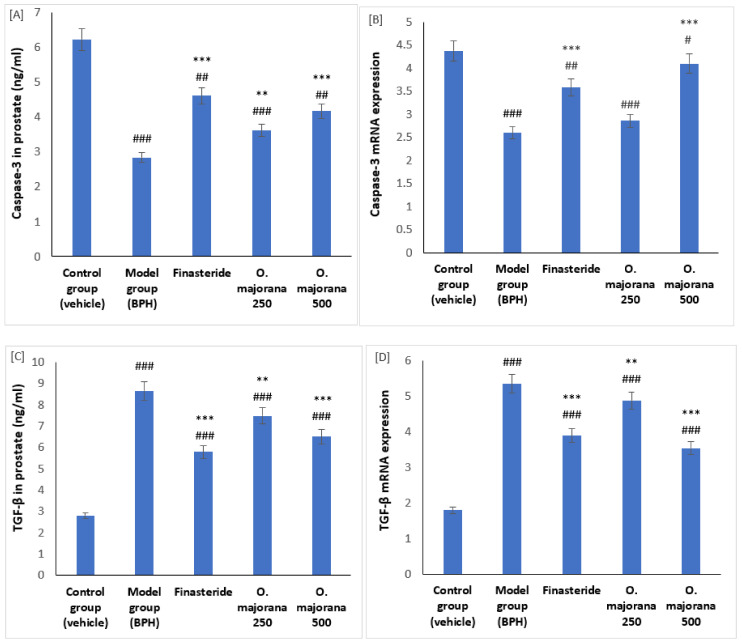
(**A**–**D**): Effect of *O. majorana* on mRNA and protein of Caspase-3 (**A**,**B**) and TGF-β1 (**C**,**D**) in prostatic tissue: (**A**) quantitative PCR of Caspase-3 mRNA, (**B**) ELISA of Caspase-3 (**C**) quantitative PCR of TGF-β1 mRNA, and (**D**) ELISA of TGF-β1 in prostatic tissue. Data are expressed as means ± SD (*n* = 8). ^###^ *p* < 0.001, ^##^ *p* < 0.01 and ^#^ *p* < 0.05 vs. control, *** *p* < 0.001 and ** *p* < 0.01 vs. BPH (model) group.

Results showed that both mRNA and protein levels of caspase-3 were significantly reduced in rats with BPH compared to control rats (*p* < 0.001). However, BPH rats treated with *O. majorana* at doses of 250 and 500 mg/kg/day showed a significant increase in levels of mRNA and protein of Caspase-3 compared to rats of both BPH and the finasteride groups as measured by quantitative PCR and ELISA immunoassay techniques (Figure 5A,B). In addition, mRNA and protein levels of TGF-β1 were identified in control, *O. majorana* BPH-treated, and non-treated rats. The BPH group showed a significant increase in the prostatic levels of TGF-β1 mRNA and protein compared to the control group (Figure 5C,D). When BPH rats were treated with *O. majorana* at doses of 250 and 500 mg/kg/day, a significant reduction in the quantitative expression of TGF-β1 mRNA and protein was identified compared to the BPH group (Figure 5C,D).

### 3.7. The Effect of O. majorana on Prostate Histological Structure

#### 3.7.1. H&E Stain

**Figure 6:**
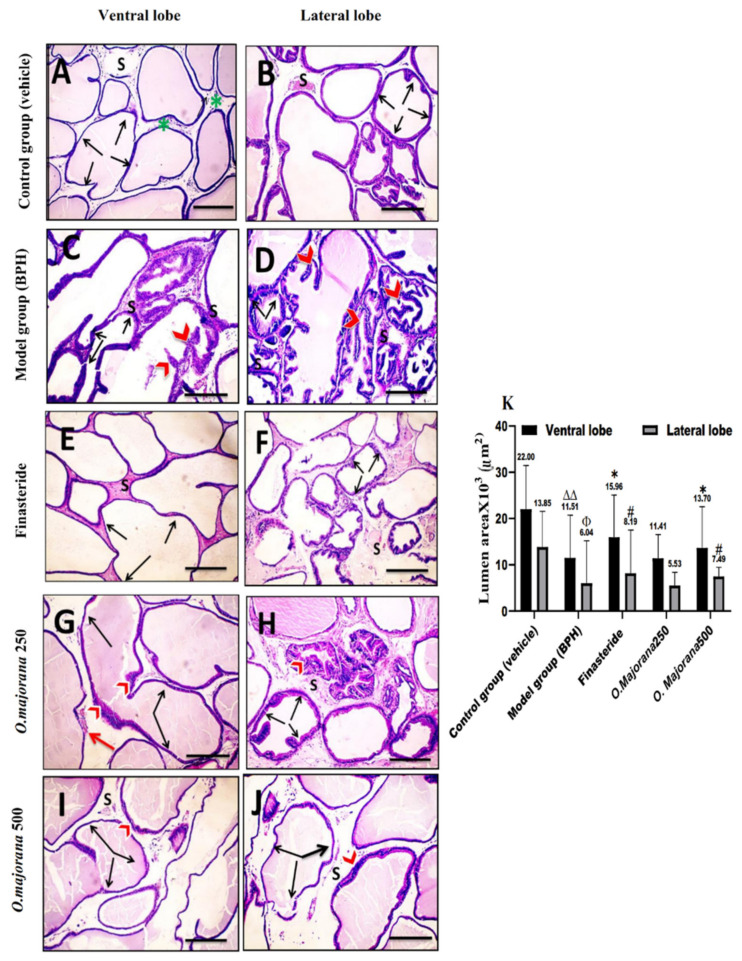
(**A**–**K**): Histological changes in prostate lobes (H&E-stained sections): Acinar luminal area. Microscopic pictures representing sections of comparable regions in ventral and lateral lobes of the control group (**A**,**B**), (BPH) group (**C**,**D**), finasteride group (**E**,**F**), *O. majorana* 250 group (**G**,**H**), and *O. majorana* 500 group (**I**,**J**). Connective tissue stroma (S) between the secretory acini, inflammatory cells infiltration (green asterisk), acinar folding (red arrowheads), dilated blood vessels (**red arrows**), and bidirectional arrows represent the acinar luminal area. Magnification, ×100. Scale bar, 100 μm. (**K**) A graph showing the acinar luminal area. Data are expressed as mean ± SD (*n* = 8). * *p* < 0.05 versus BPH (model) group and ^∆∆^ *p* < 0.01 vs. control for ventral lobe, ^#^ *p* < 0.05 vs. BPH (model) group and Φ *p* < 0.05 vs. control for lateral lobe.

**Figure 7:**
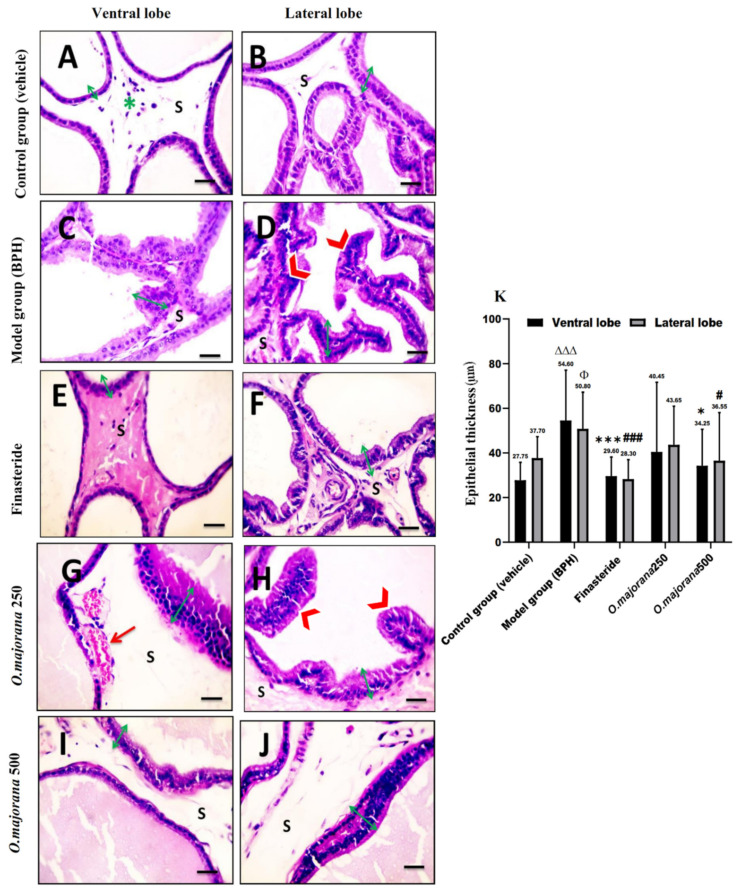
(**A**–**K**): Histological changes in prostate lobes (H&E-stained sections): Epithelial height of the prostatic tissue. Microscopic pictures representing sections of comparable regions in ventral and lateral lobes of the control group (**A**,**B**), (BPH) group (**C**,**D**), finasteride group (**E**,**F**), *O. majorana* 250 group (**G**,**H**), and *O. majorana* 500 group (**I**,**J**). Connective tissue stroma (S) between the secretory acini, inflammatory cells infiltration (**green asterisk**), acinar folding and epithelial thickening (**red arrowheads**), dilated blood vessels (**red arrows**), and green **↔** arrows represent the prostatic epithelium height. Magnification, ×400. Scale bar, 50 μm. (**K**) A graph presenting the epithelial thickening. Data are expressed as mean ± SD (*n* = 8). * *p* < 0.05, *** *p* < 0.001 vs. BPH (model) group and ^∆∆∆^ *p* < 0.001 versus control for ventral lobe, ^#^ *p* < 0.05, ^###^ *p* < 0.001 vs. BPH (model) group and Φ *p* < 0.05 versus control for lateral lobe.

The vehicle/control group revealed tight tubular prostatic glands with thin-walled acini containing pale eosinophilic secretions in ventral lobe regions (Figure 6A and Figure 7A). Lateral prostatic lobes acini were smaller than those of the ventral lobes with a more pronounced degree of folding alveoli and presented a light eosinophilic secretion (Figure 6B and Figure 7B). The acini in both lobes were surrounded by connective tissue stroma infiltrated with few inflammatory cells. The BPH/model group showed glandular hypertrophy with numerous acini, thick epithelial lining projecting into the acinar cavities as papillary fronds, nuclear stratification, and reduced size of the acinar lumen in both ventral (Figure 6C and Figure 7C) and lateral lobes acini (Figure 6D and Figure 7D). In the finasteride-treated group, ventral and lateral prostatic lobes showed reduced epithelial thickening and increased glandular luminal area (Figure 6E,F and Figure 7E,F) of most acini. The *O. majorana* 250 group showed non-remarkable histological difference compared to the BPH/model group, especially in lateral lobes with the presence of dilated blood vessels in the connective tissue stroma (Figure 6G,H and Figure 7G,H). However, the *O. majorana* 500 group revealed an evident increase in the acinar luminal area and reduction in the height of acinar epithelial lining with less polyp formation (Figure 6I,J and Figure 7I,J) in both ventral and lateral lobes.

Statistical analysis of acinar epithelial height (Figure 7K) showed significantly increased epithelial height in acini of ventral and lateral lobes in the BPH/model group relative to the control group. When compared to BPH (model group), the finasteride-treated group and *O. majorana* 500 group had significantly less acinar epithelial height in both ventral and lateral lobes (*p* < 0.001 and *p* < 0.05, respectively). The *O. majorana* 250 group showed an insignificant reduction in the acinar epithelial height relative to BPH/model group (*p ≥* 0.05). The acinar luminal area of ventral and lateral lobes showed a significant reduction in BPH/model group (*p* < 0.001 and *p* < 0.05, respectively) relative to the control group (Figure 6K). When compared to the BPH/model group, the finasteride-treated group and *O. majorana* 500 group revealed significant widening of the acinar lumen in glands of both ventral and lateral lobes (*p* < 0.05), which was insignificant (*p* ≥ 0.05) with *O. majorana* 250 group.

#### 3.7.2. Masson’s Trichrome Stain

**Figure 8:**
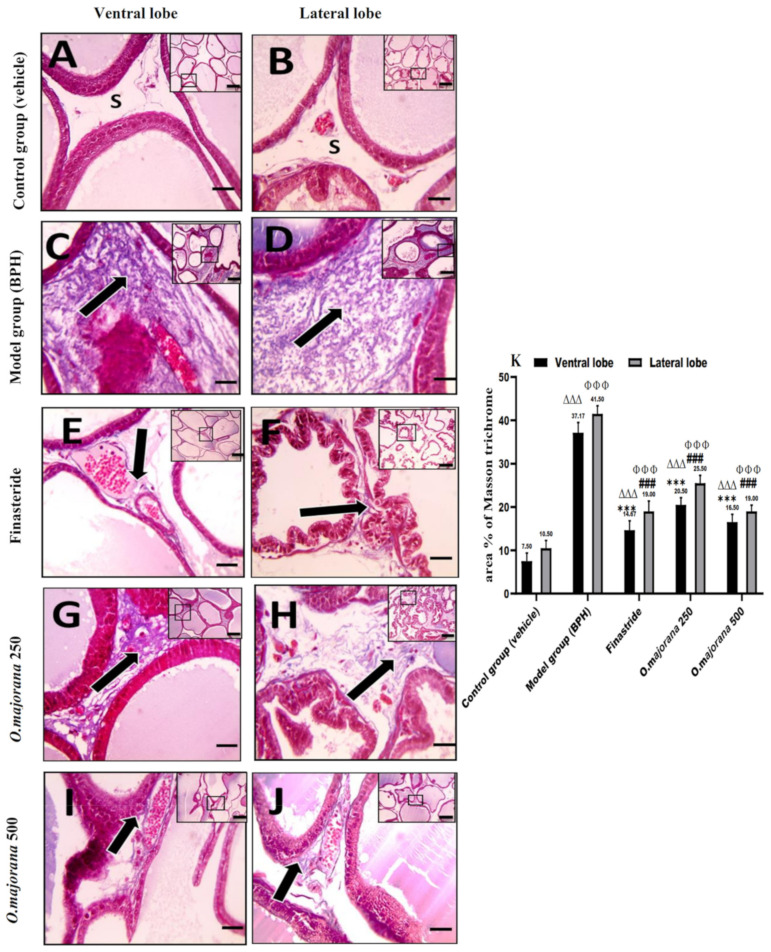
(**A**–**K**): Histological changes in prostate lobes (Masson’s trichrome-stained sections): Microscopic pictures representing sections of comparable regions in ventral and lateral lobes of the control group (**A**,**B**), (BPH) group (**C**,**D**), finasteride group (**E**,**F**), *O. majorana* 250 group (**G**,**H**), and *O. majorana* 500 group (**I**,**J**). CT stroma between the acini (**S**) and collagen fibers deposition (**black arrows**). X: 100 bar 100 μm (low magnification) and X: 400 bar 50 μm (high magnification). (**K**) A graph showing collagen fiber means area percentage in the experimental groups. Data are expressed as mean ± SD (*n* = 8). *** *p* < 0.001 vs. BPH (model) group and ^∆∆∆^ *p* < 0.001 versus control for ventral lobe, ^###^ *p* < 0.001 vs. BPH (model) group and ^ΦΦΦ^ *p* < 0.001 versus control for lateral lobe.

Control group sections stained with Masson’s trichrome displayed normal CT stroma with few collagen fibers in the between acini of the ventral and lateral prostatic lobes (Figure 8A,B), whereas the collagen fiber content of CT stroma in the (BPH) group was significantly higher than (*p* < 0.001) the control group (Figure 8C,D,K). When compared to the (BPH) group, the finasteride and *O. majorana* 500-treated groups presented significantly (*p* < 0.001) less amounts of deposited collagen fibers between secretory acini (Figure 8I,J and Figure 8E,F,K). The *O. majorana* 250 group showed a non-significant increase in deposited stromal collagen fibers relative to the finasteride group (Figure 8G,H), which was significantly less than the (BPH) group (*p* < 0.001, Figure 8K).

### 3.8. α-SMA and TGF-B1 Expression in Prostate Lobes

#### 3.8.1. α-SMA Immunostaining

**Figure 9:**
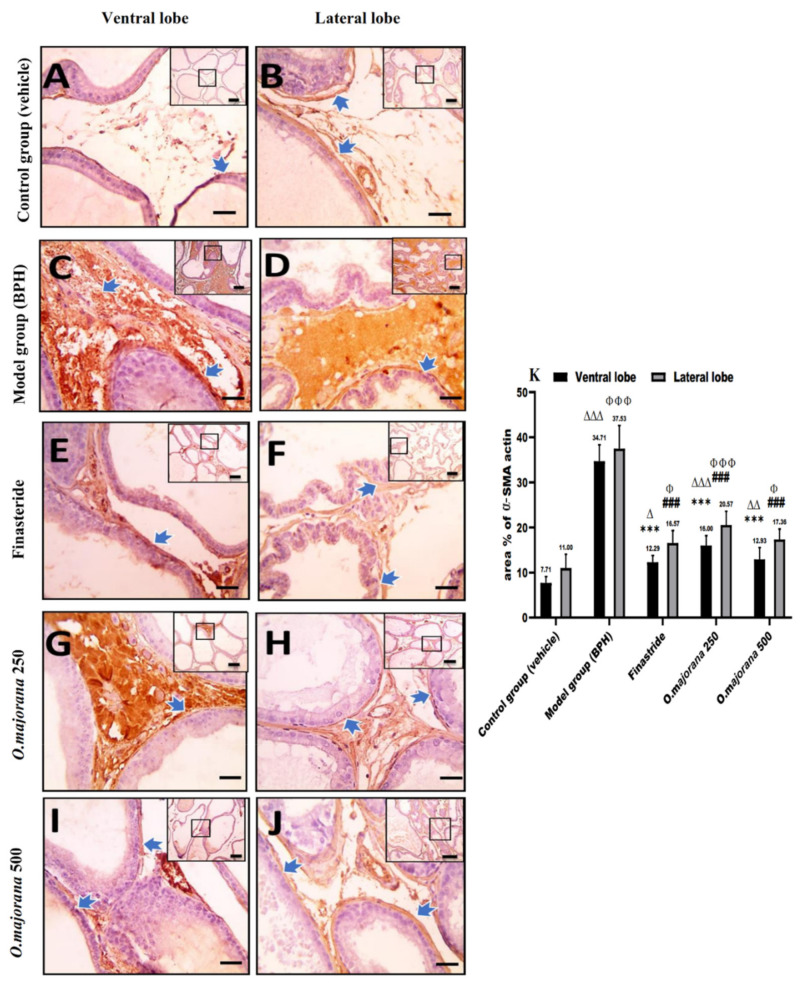
(**A**–**K**): Histological changes in prostate lobes (immunostained prostate sections against α-SMA actin): Microscopic pictures sections of comparable regions in ventral and lateral lobes of the control group (**A**,**B**), (BPH) group (**C**,**D**), finasteride group (**E**,**F**), *O. majorana* 250 group (**G**,**H**), and *O. majorana* 500 group (**I**,**J**). Blue arrows indicate positive staining. Immunohistochemistry (IHC) counterstained with Mayer’s hematoxylin. Low magnification X: 100 bar 100 μm and high magnification X: 400 bar 50 μm. (**K**) A graph showing the mean area % of actin in the experimental groups. Data are expressed as mean ± SD (*n* = 8). *** *p* < 0.001 versus BPH (model) group and ^∆^ *p* < 0.05, ^∆∆^ *p* < 0.01 ^∆∆∆^ *p* < 0.001 versus control for ventral lobe, ^###^ *p* < 0.001 versus BPH (model) group and ^Φ^ *p* < 0.05, ^ΦΦΦ^ *p* < 0.001 versus control for lateral lobe.

Prostatic sections stained with anti-αSMA, a specific stromal myofibroblasts’ marker, exhibited positive expression in stromal cells around prostatic acini (Figure 9A,B). When compared to the control group, the (BPH) group expressed significantly higher α-SMA levels (*p* < 0.001, Figure 9K) around the acini of both ventral and lateral prostatic lobes (Figure 9C,D). Finasteride and *O. majorana* 500 groups showed a marked reduction in the α-SMA-positive brown stain observed in smooth muscles encircling the acini (Figure 9E,F,I,J) when compared with the BPH group. Similarly, the *O. majorana* 250 group (Figure 9G,H) showed a significant reduction in the α-SMA-positive brown stain when compared with the BPH group (*p* < 0.001; Figure 9K).

#### 3.8.2. TGF-B1 Immunostaining

**Figure 10:**
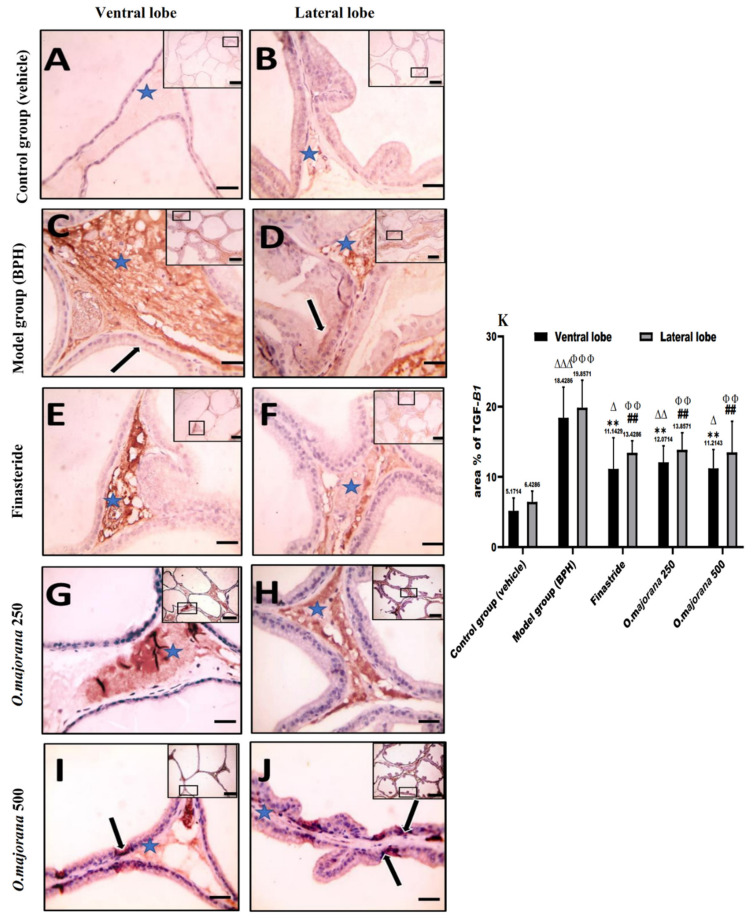
(**A**–**K**)**:** Histological changes in prostate lobes (immunostained prostate sections against (TGF-*B1*)): Microscopic pictures representing sections of comparable regions in ventral and lateral lobes of the control group (**A**,**B**), (BPH) group (**C**,**D**), finasteride group (**E**,**F**), *O. majorana* 250 group (**G**,**H**), and *O. majorana* 500 group (**I**,**J**). Blue asterisk points to positive interstitial stroma, and black arrows demonstrate positive epithelial immunoreactivity. IHC counterstained with Mayer’s hematoxylin. X: 100 bar 100 μm (low magnification) and X: 400 bar 50 μm (high magnification). (**K**) A graph showing mean area % of actin in the studied groups. Data are expressed as mean ± SD (*n* = 8). ** *p* < 0.01 versus BPH (model) group and ^∆^ *p* < 0.05, ^∆∆^ *p* < 0.01 ^∆∆∆^ *p* < 0.001 versus control for ventral lobe, ^##^ *p* < 0.01versus BPH (model) group and ^ΦΦ^ *p* < 0.01, ^ΦΦΦ^ *p* < 0.001 versus control for lateral lobe.

Immunostained prostatic sections with anti-TGF-B1 showed negative staining of the epithelial cells with mild staining of the interstitial tissue in the control group (Figure 10 A,B). Interstitial tissue and epithelial cells in the (BPH) group’s ventral and lateral prostatic lobes strongly expressed positive brown staining (Figure 10C,D), which was significant relative to the control group (*p* < 0.001, Figure 10K). Finasteride and *O. majorana* 500 groups’ sections (Figure 10E,F,I,J) showed a sporadic positive brown staining in acinar epithelial cells with mild positive brown staining of the interstitial tissue, which was significant (*p* < 0.001, Figure 10K) relative to the BPH group. Sections from the *O. majorana* 250 group displayed a moderately positive expression of anti-TGF-B1 in the interstitial tissue (Figure 10G,H), which was significantly less relative to the BPH group (*p* < 0.001, Figure 10K).

### 3.9. Apoptotic and Proliferative Indices

**Figure 11:**
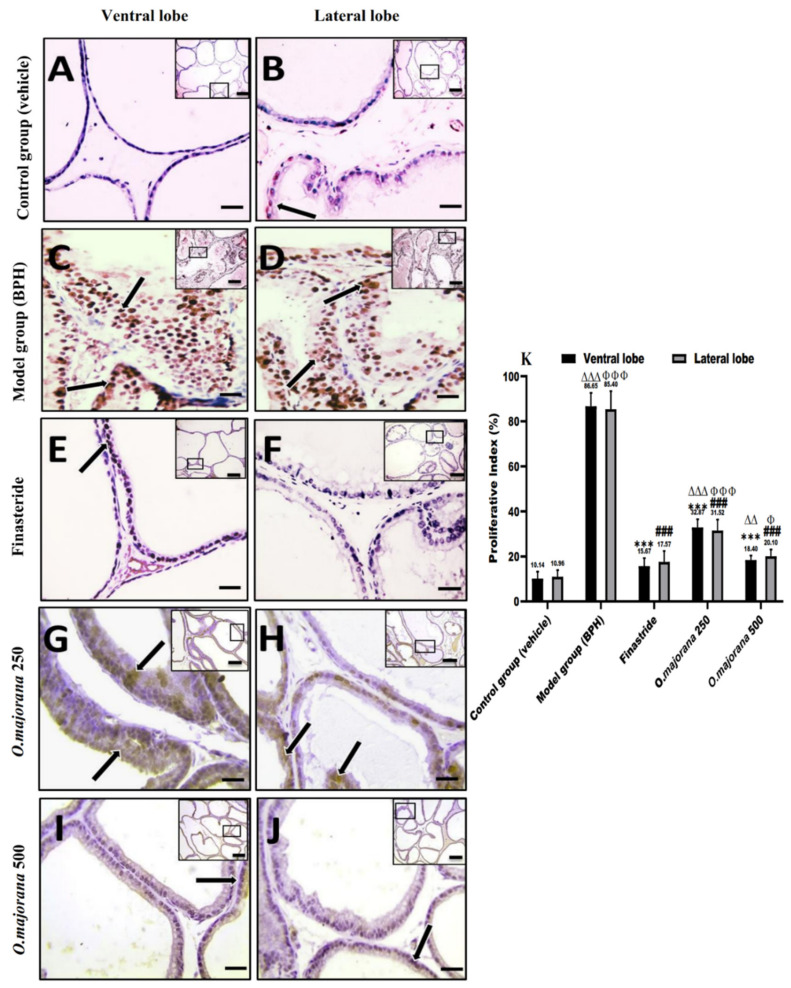
(**A**–**K**)**:** Histological changes in prostate lobes (immunostained prostate sections against (PCNA)): Microscopic pictures representing sections of comparable regions in ventral and lateral lobes of the control group (**A**,**B**), (BPH) group (**C**,**D**), finasteride group (**E**,**F**), *O. majorana* 250 group (**G**,**H**), and *O. majorana* 500 group (**I**,**J**). Black arrows demonstrate positive epithelial immunoreactivity. IHC counterstained with Mayer’s hematoxylin. X: 100 bar 100 μm (low magnification) and X: 400 bar 50 μm (high magnification). (**K**) A graph showing the proliferative index. Data represent the mean percentage of cells that showed positive immunoreactivity ± SD (*n* = 8). *** *p* < 0.001 vs. BPH (model) group and ^∆∆^ *p* < 0.01, ^∆∆∆^ *p* < 0.001 vs. control for ventral lobe, ^###^ *p* < 0.001 vs. BPH (model) group and ^Φ^ *p* < 0.05, ^ΦΦΦ^ *p* < 0.001 vs. control for lateral lobe.

**Figure 12:**
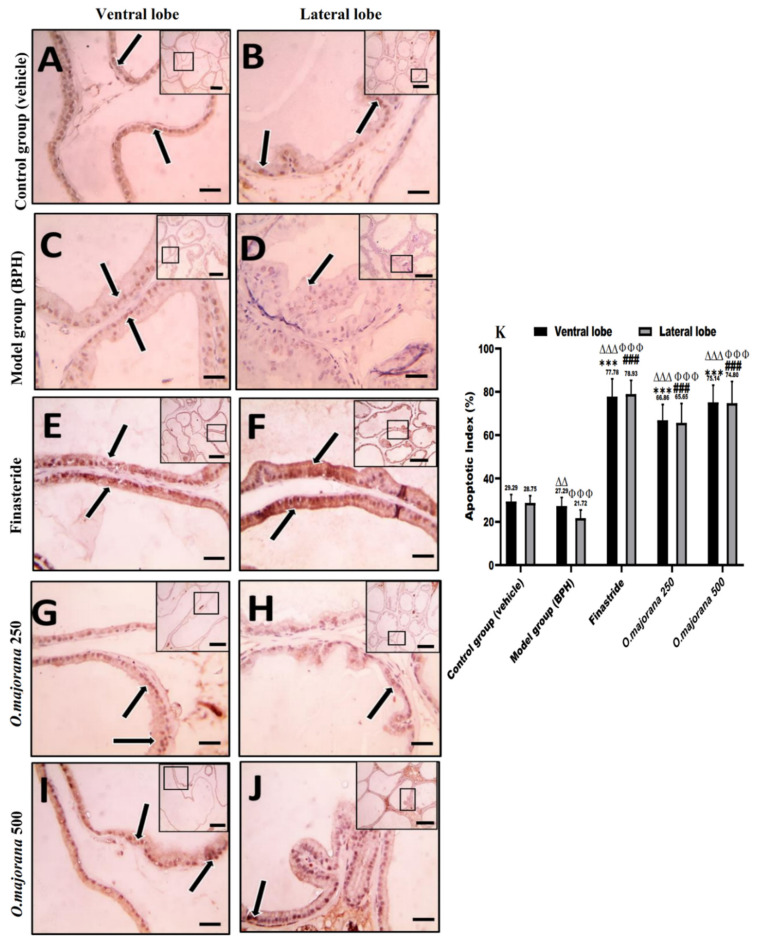
(**A**–**K**)**:** Histological changes in prostate lobes (immunostained prostate sections against (caspase-3)): Microscopic pictures representing sections of comparable regions in ventral and lateral lobes of the control group (**A**,**B**), (BPH) group (**C**,**D**), finasteride group (**E**,**F**), *O. majorana* 250 group (**G**,**H**), and *O. majorana* 500 group (**I**,**J**). Black arrows demonstrate positive epithelial immunoreactivity. IHC counterstained with Mayer’s hematoxylin. X: 100 bar 100 μm (low magnification) and X: 400 bar 50 μm (high magnification). (**K**) A graph showing the apoptotic index. Values represent the mean percentage of cells that showed positive immunoreactivity ± SD (*n* = 8). *** *p* < 0.001 vs. BPH (model) group and ^∆∆^ *p* < 0.01, ^∆∆∆^ *p* < 0.001 vs. control for ventral lobe, ^###^ *p* < 0.001 vs. BPH (model) group and ^ΦΦΦ^ *p* < 0.001 vs. control for lateral lobe.

In normal and hyperplastic prostatic sections, proliferating cells (PCNA immunohistochemical identification) and apoptotic cells (caspase-3 immunohistochemical identification) were observed, with only a few stromal cells staining (Figure 11 and Figure 12).

Epithelial cells in the control group sections showed negative to mild nuclear staining, especially in the lateral lobe, and the mean proliferative index (PI) was 10.14 ± 3.04 and 10.96 ± 2.92 for ventral and lateral lobes, respectively (Figure 11A,B,K). Photomicrographs of the (BPH) group showed a strong positive brown expression in the epithelial cells’ nuclei of both ventral and lateral prostatic lobes (Figure 11C,D), which was significant (*p* < 0.001; Figure 11K) when compared to controls. The mild positive brown expression of PCNA in epithelial cells nuclei of finasteride and *O. majorana* 500 groups’ sections (Figure 11E,F,I,J) was significantly less relative to the BPH group (*p* < 0.001, Figure 11K). When compared to the BPH group, *O. majorana* 250 group sections (Figure 11G,H) displayed a significantly less positive nuclear expression of PCNA (*p* < 0.001, Figure 11K).

The control group exhibited moderate epithelial expression of caspase-3 in the ventral and lateral lobes, with mean apoptotic index (AI) values of 29.29 ± 3.32 and 28.75 ± 3.23, respectively (Figure 12A,B,K). Microscopic pictures of the (BPH) group (Figure 12C,D) showed sporadic positive brown cytoplasmic staining in epithelial cells compared with controls in both ventral and lateral prostatic lobes, which was significant *(p* < 0.01 and *p* < 0.001, respectively, Figure 12K). The positive brown cytoplasmic staining markedly increased in epithelial cells in the finasteride group and *O. majorana* 500 groups (Figure 12E,F,I,J, respectively) which was significant (*p* < 0.001; Figure 12K) relative to the BPH group. Positive brown cytoplasmic staining appears moderate in the *O. majorana* 250 group (Figure 12G,H), which was significant (*p* < 0.001, Figure 12K) when compared with the BPH group.

**Figure 13:**
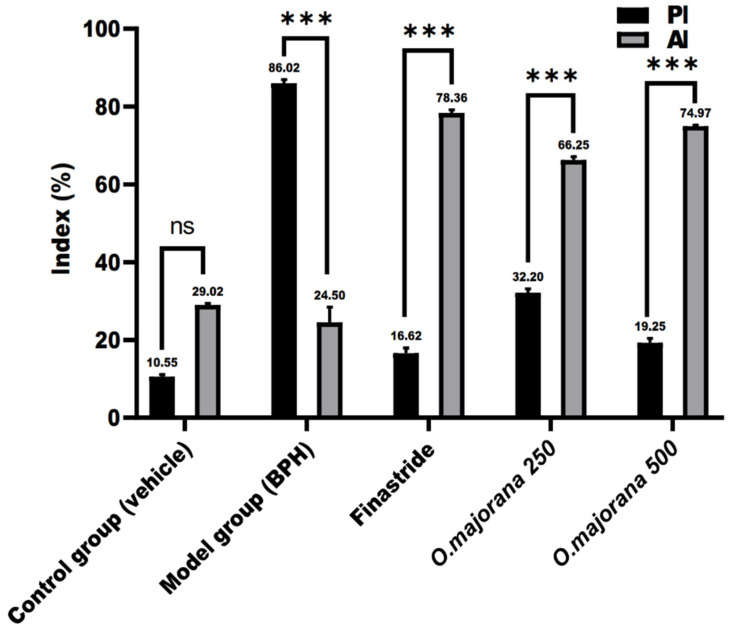
A graph showing the proliferative index (PI) and apoptotic index (AI) in the experimental groups. Values represent the mean percentage of cells that showed positive immunoreactivity ± SD (*n* = 8). *** *p* < 0.001 of PI versus AI in individual groups. ns = non-significant.

Considering the absence of significant variation between ventral and lateral prostatic lobes among the experiment groups, the proliferative index (PI) and the apoptotic index (AI) of prostatic sections of each experimental group were compared to other groups without lobe discrimination, as shown in Figure 13. The normal control group’s PI and AI (10.55 ± 0.57 and 29.02 ± 0.38, respectively) were insignificant (*p* = 0.15) with a mean difference (−18.47 ± 0.49). The BPH group had a significantly (*p* < 0.001) higher PI than AI (86.02 ± 0.88 and 24.50 ± 3.93, respectively), with a mean difference (61.52 ± 2.85). The mean difference between PI and AI in finasteride, *O. majorana* 250, and *O. majorana* 500 groups was (−61.74 ± 1.11, −34.05 ± 0.91, and −55.72 ± 0.87, respectively), which was significant (*p* < 0.001).

### 3.10. γ H2Ax and PARP1 Expression in Prostate Lobes

#### 3.10.1. γ H2Ax Expression in Prostate Lobes

**Figure 14:**
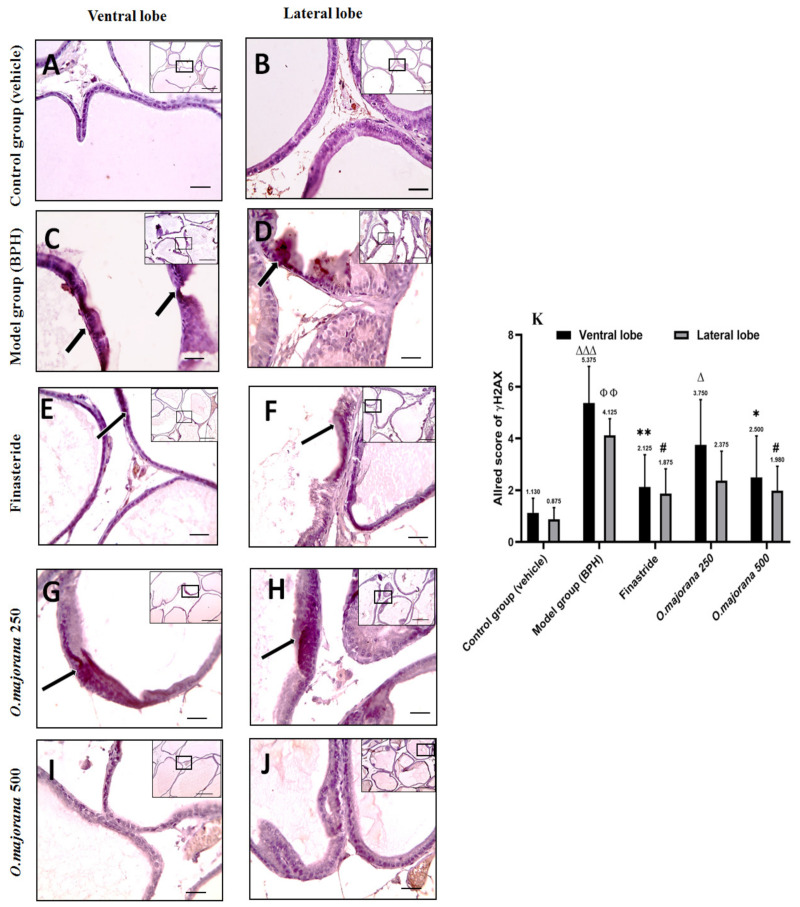
(**A**–**K**)***:*** Histological changes in prostate lobes (immunostained prostate sections against (γH2Ax): Microscopic pictures representing sections of comparable regions in ventral and lateral lobes of the control group (**A**,**B**), (BPH) group (**C**,**D**), finasteride group (**E**,**F**), *O. majorana* 250 group (**G**,**H**) and *O. majorana* 500 group (**I**,**J**). **Black arrows** demonstrate positive epithelial immunoreactivity. IHC counterstained with Mayer’s hematoxylin. X: 100 bar 100 μm (low magnification) and X: 400 bar 50 μm (high magnification). (**K**) A graph showing the Allred scoring system. Values are expressed as mean ± SD (*n* = 8). 0–1 (negative), 2–3 (mild), 4–6 (moderate) and 7–8 (strong). * *p* < 0.05, ** *p* < 0.01 vs. BPH (model) group and ^∆^ *p* < 0.05, ^∆∆∆^ *p* < 0.001 vs. control for ventral lobe, ^#^ *p* < 0.05 vs. BPH (model) group and ^ΦΦ^ *p* < 0.01 vs. control for lateral lobe.

Epithelial cells in the control group sections showed negative staining, especially in the lateral lobe, and the Allred score was (1.13 ± 0.56, 0.88 ± 0.45) for ventral and lateral lobes, respectively (Figure 14A,B,K). Photomicrographs of the (BPH) group showed multifocal areas of positive brown cytoplasmic staining in epithelial cells in both ventral and lateral prostatic lobes (Figure 14C,D), with an Allred score (5.38 ± 1.41, 4.13 ± 0.64) that was significant (*p* < 0.001 and *p* < 0.01; Figure 14K) respectively when compared to controls. The mild positive brown cytoplasmic expression of γH2Ax in epithelial cells of finasteride and *O. majorana* 500 groups’ sections (Figure 14E,F,I,J) was significantly less relative to the BPH group (Figure 14K). When compared to the BPH group, *O. majorana* 250 group sections (Figure 14G,H) displayed moderate positive brown cytoplasmic staining in epithelial cells, especially in the ventral lobe with an Allred score (2.50 ± 1.60) (Figure 14K).

#### 3.10.2. PARP1 Expression in Prostate Lobes

**Figure 15:**
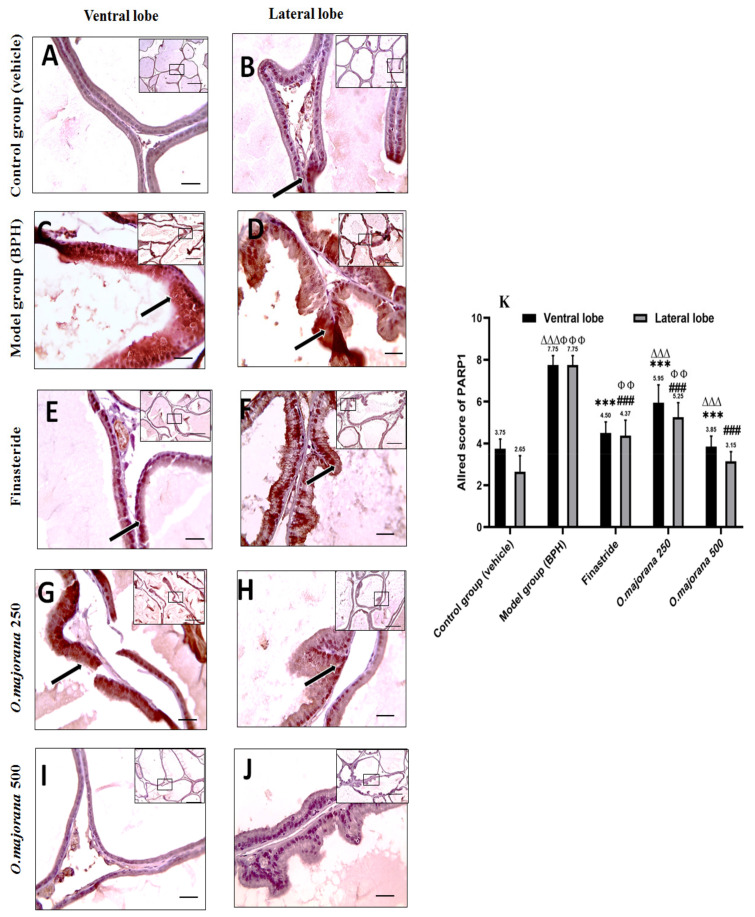
(**A**–**K**)**:** Histological changes in prostate lobes (immunostained prostate sections against (PARP1)): Microscopic pictures representing sections of comparable regions in ventral and lateral lobes of the control group (**A**,**B**), (BPH) group (**C**,**D**), finasteride group (**E**,**F**), *O. majorana* 250 group (**G**,**H**) and *O. majorana* 500 group (**I**,**J**). Black arrows demonstrate positive epithelial immunoreactivity. IHC counterstained with Mayer’s hematoxylin. X: 100 bar 100 μm (low magnification) and X: 400 bar 50 μm (high magnification). (**K**) A graph showing the Allred scoring system. Values are expressed as mean ± SD (*n* = 8). 0–1 (negative), 2–3 (mild), 4–6 (moderate) and 7–8 (strong). *** *p* < 0.001 vs. BPH (model) group and ^∆∆∆^ *p* < 0.001, vs. control for ventral lobe, ^###^ *p* < 0.001 vs. BPH (model) group and ^ΦΦ^ *p* < 0.01, ^ΦΦΦ^ *p* < 0.001 vs. control for lateral lobe.

Epithelial cells in the control group sections showed mild positive staining, especially in the lateral lobe, and the Allred score was (3.75 ± 0.46, 2.65 ± 0.76) for ventral and lateral lobes, respectively (Figure 15A,B,K). Photomicrographs of the (BPH) group showed diffuse strong positive brown cytoplasmic staining in epithelial cells in both ventral and lateral prostatic lobes (Figure 15C,D), with an Allred score (7.75 ± 0.45) in both lobes that was significant (*p* < 0.001; Figure 15K) when compared to controls. The mild positive brown cytoplasmic expression of PARP1 in epithelial cells of *O. majorana* 500 groups’ sections (Figure 15I,J) was significantly less in both lobes relative to the BPH group (*p* < 0.001; Figure 15K). When compared to the BPH group, *O. majorana* 250 group sections (Figure 15G,H) displayed moderate positive brown cytoplasmic staining in epithelial cells, especially in the ventral lobe with an Allred score (5.95 ± 0.85) (Figure 15K).

## 4. Discussion

**Figure 16:**
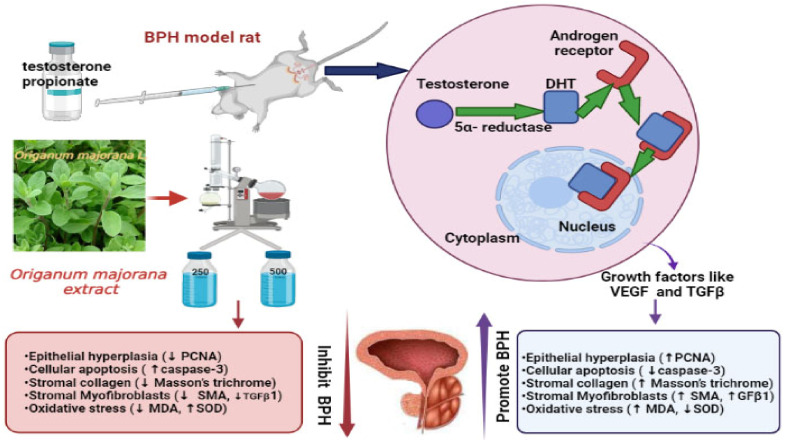
Graphical abstract.

BPH is a progressive illness accompanied by benign prostate enlargement (BPE) and lower urinary obstruction symptoms [58]. Therapeutic approaches for BPH include surgical and medicinal; however, medicinal therapy showed incomplete effective cure for BPH [59] in addition to their adverse effects [60], and the surgical approach is frequently associated with multiple complications, particularly in older men [61]. In this study, we are shedding light on the antiproliferative, apoptotic, and antioxidative properties of *O. majorana* against BPH for the first time using TP-induced BPH rats after castration to mask the effect of endogenous hormones and evaluate its impact on the prostate through histological, immunohistochemical, and biochemical approaches. The results of the current study are summarized in Figure 16.

BPH was distinguished by histological examination as hyperplasia and desquamation of the acinar epithelial cell lining, inflammatory cell infiltration, and papillary projections, as well as elevated prostate weight and prostate index, which are consistent with previous findings [62]. Our findings supported our working hypothesis, demonstrating that *O. majorana* extract alleviated testosterone-induced BPH rats by lowering prostatic index, reducing serum DHT levels, reducing prostatic MDA content and DNA damage, elevating prostatic SOD content, mitigating the histopathological changes of BPH, increasing the apoptotic index while lowering the proliferative index, and reducing stromal markers, α-SMA, and TGF-*B1* expression. The *O. majorana* 500-treated group outperformed the *O. majorana* 250-treated group. Furthermore, the findings reported in the *O. majorana* 500-treated groups were close to those gained with finasteride treatment.

The correlation between oxidative stress and BPH was previously reported with a significant reduction in serum and prostate antioxidant levels in BPH rats [5,33,58]. Previous studies reported the antioxidant activity of *O. majorana* against renal and hepatic injuries [36,37,63] and attributed this property to the effect of its secondary metabolites, particularly flavonoids, in preventing oxidative cell damage through their water-soluble effects [64], in addition to its inhibiting effect on the DPPH (2, 2-diphenyl-1-picrylhydrazyl) [38]. These reports are consistent with our data considering reduced prostatic MDA content and elevated prostatic SOD content with *O. majorana*. Increased oxidative stress is associated with marked DNA damage in BPH patients. Management of oxidative stress and DNA repair is suggested to be a useful strategy in disease therapy and prevention [65]. In the present study, increased immunostaining of γ-H2AX and PARP, as well as significant increase in tissue 8-OH-dG in the BPH group, reflected DNA damage. However, this situation was ameliorated by *O. majorana* treatment.

The Nrf2/HO-1 pathway has been widely studied with regard to its role in combating inflammation and fibrosis [66]. In BPH, Nrf2 and its target genes have been found to be deficient and insufficient for antioxidant response [67]. Nrf2 regulates the expression of key protective elements in BPH [68]. On the other hand, activation of Nrf2 blocks TGF-β signaling and suppresses inflammation, thereby restraining prostatic epithelial-mesenchymal transition in BPH [69]. Consistently, in this study, the antioxidative potency of *O. majorana* is associated with increased levels of Nrf2 and HO-1 in BPH rats treated with *O. majorana* at doses of 250 and 500 mg.

In addition, our data reported that *O. majorana* at respective doses of 250 and 500 mg/kg/day significantly reduced the cellular DNA damage and increased both catalase enzyme (CAT) and total antioxidant activity (TAC) in BPH rats, suggesting the antioxidant effect of *O. majorana* against cellular severity of BPH. The production of ROS cellular free radicals causes DNA base modifications such as a hydroxyl radical that can attack DNA to form 8-hydroxyguanine (8-OH-dG) [70,71,72,73,74], increasing the probability of prostate carcinogenesis [70,71,72,73,74].

Previous studies reported that *O. majorana* extracts have an antiproliferative impact on a variety of cancer cell lines [24,75,76,77,78,79,80] and illustrated actions achieved by its compounds, including: detained cell cycle, cell migration, angiogenesis inhibition, apoptosis, telomerase activity modulation, and ROS consequences, which end by suppression of malignant cell proliferation and invasion [81,82,83]. These reports are in agreement with our data, which showed a significant increase in AI concurrent with a reduction in the PI in the *O. majorana* 500-treated group, followed by the *O. majorana* 250-treated group.

The role of caspases in prostate regression following castration was previously proposed [84]. Moreover, Omezzine et al. [85] documented a relation between inhibited apoptotic cell death pathway by testosterone in the ventral prostate and reduced both caspase-3 and -6 mRNA production. A recent study also observed the exhibited induction of BPH together with reduced caspase activity [63]. The resistance to induce cellular apoptosis results in increased severity of BPH and might progress to prostate cancer. Thus, targeting apoptosis in BPH is of great importance. These findings are in accordance with the results of the present study that demonstrated *O. majorana*’s apoptotic effect, given that it reduces PCNA immunoreactivity while increasing caspase-3 mRNA, protein levels, and immunoreactivity.

Our data of reduced mean area% of collagen, α-SMA, and TGF-β1 immunoreactivity in the stroma of *O. majorana*-treated groups are consistent with lower PI and higher AI and align with the previously documented correlation between stromal hyperplasia and induced epithelial growth in the pathogenesis of BPH [86]. The immunolocalization results were further reinforced by results of TGF- β1 mRNA and protein levels. Myofibroblasts and their precursor fibrocyte overgrowth were found to directly and/or indirectly induce prostatic hyperplasia through a local mesenchymal epithelial interaction [87]. Previous research also linked the decrease in TGF-ß1 expression to inhibition of stromal extracellular matrix (ECM) deposition and hyperplastic prostatic growth [88,89]. TGF-β is a central mediator of fibrogenesis [90]. The cellular expression of TGF-β1 in large amounts in BPH prostate tissues was found to induce global changes in DNA methylation during the epithelial-to-mesenchymal transition state [48,91].

## 5. Conclusions

Collectively, our study based upon molecular PCR, biochemical, morphological, and immunohistochemical reactivity analysis significantly supports the efficiency of *O. majorana* extract, in a dose-dependent manner, as an adjuvant treatment for PBH. This was evidenced by downregulation of DHT, reduced PW and PI, elevated proapoptotic Caspase-3, SOD, CAT, and TAC levels and lowered proinflammatory TGF-β1, cellular DNA damage, and MDA, and regression of epithelial and stromal hyperplasia in experimental BPH rats through its antioxidative, antiproliferative, and proapoptotic properties. We suggest that *O. majorana* extract can establish a balance between proliferation and apoptosis in BPH.

## Figures and Tables

**Table 1:** List of primers sequences used in real-time PCR analysis.

Gene	Primer Sequences
*GAPDH*	For 5′- CCTCTGACTTCAACAGCGAC-3 Rev 5′-TCCTCTTGTG CTCTTGCTGG-3′
Caspase-3	For 5′-GGTATTGAGACAGACAGTGG-3′ Rev 5′-CATGGGATCTGTTTCTTTGC-3′
TGF-β1	For 5′-CAATTCCTGGCGATACCTCAG-3″ Rev 5′-GCACAACTCCGGTGACATCAA-3

**Table 2:** Antibody specifics and functions.

Primary Antibody	Function	Manufacturer (Cat. No.)	Host Species	Dilution
γH2AX	A marker for DNA double-strand breaks	phospho-S139, Abclonal, Wuhan, China, AP0099)	Rabbit polyclonal	1:100
PARP	Sensor of DNA damage	Abclonal, Wuhan, China, A0942	Rabbit polyclonal	1:100
PCNA	Cell proliferation marker	Santa Cruz Biotechnology, Inc., Santa Cruz, CA, USA (Sc-56)	Mouse monoclonal	1:100
Caspase-3	Proapoptotic marker	Abcam (ab184787)	Rabbit monoclonal	1:100
TGF-β1	Apoptosis, marker for Fibrogenesis. extracellular matrix formation, and morphogenesis	Santa Cruz Biotechnology, Inc. (SC-130348)	Mouse monoclonal	1:100
α-SMA	Marker for smooth muscle fibers surrounding the acini	Biolegend (MMS-466S)	Rabbit polyclonal	1:100

**Table 3:** Rats’ body weight and prostate parameters.

Groups	Gain in Body Weight (g)	Prostate Weight (mg)	% Inhibition Prostate Weight	Prostate Index × 10^−3^	% Inhibition Prostate Index
Control group(vehicle)	38.25 ± 4.65	380.59 ± 13.58 ^a^	-	1.14 ± 0.02 ^a^	-
Model group (BPH)	21.00 ± 3.16 *	912.50 ± 85.39 ^b^	-	2.85 ± 0.26 ^b^	-
Finasteride	12.25 ± 2.85 *	600.02 ± 81.64 ^c^	58.75	1.95 ± 0.28 ^c^	52.29
*O. majorana 250*	20.00 ± 1.63 *	812.50 ± 49.30 ^b,d^	18.80	2.59 ± 0.44 ^b,d^	15.03
*O. majorana 500*	15.75 ± 8.42 *	637.50 ± 47.87 ^c,e^	51.70	2.04 ± 0.16 ^c,e^	47.43

The values are described as mean ± SD (*n* = 8 animals/group). * *p* < 0.05 vs. control. Different letters in a column represent the statistical difference (*p* < 0.05).

## Data Availability

Data is contained within the article.

## Supplementary Materials

The following are available online at https://www.mdpi.com/article/10.3390/antiox11061149/s1, Figure S1: Effect of *O. majorana* (500 mg/kg/day) on kidney and liver function indices when administrated to the normal control group. Figure S2: Microscopic pictures of H&E-stained hepatic and renal sections from the control group and control treated with *O. majorana* 500 mg/kg/day.

## References

[1] Gacci M., Corona G., Salvi M., Vignozzi L., McVary K.T., Kaplan S.A., Roehrborn C.G., Serni S., Mirone V., Carini M. (2012). A systematic review and meta-analysis on the use of phosphodiesterase 5 inhibitors alone or in combination with α-blockers for lower urinary tract symptoms due to benign prostatic hyperplasia. Eur. Urol..

[2] El-Sherbiny M., El-Shafey M., El-Din El-Agawy M.S., Mohamed A.S., Eisa N.H., Elsherbiny N.M. (2021). Diacerein ameliorates testosterone-induced benign prostatic hyperplasia in rats: Effect on oxidative stress, inflammation and apoptosis. Int. Immunopharmacol..

[3] Atawia R.T., Tadros M.G., Khalifa A.E., Mosli H.A., Abdel-Naim A.B. (2013). Role of the phytoestrogenic, pro-apoptotic and anti-oxidative properties of silymarin in inhibiting experimental benign prostatic hyperplasia in rats. Toxicol. Lett..

[4] Xu D.-H., Wang L.-H., Mei X.-T., Li B.-J., Lv J.-L., Xu S.-B. (2014). Protective effects of seahorse extracts in a rat castration and testosterone-induced benign prostatic hyperplasia model and mouse oligospermatism model. Environ. Toxicol. Pharmacol..

[5] Cai H., Zhang G., Yan Z., Shang X. (2018). The effect of Xialiqi capsule on testosterone-induced benign prostatic hyperplasia in rats. Evid. Based Complement. Alternat. Med..

[6] Yuan J., Liu Y., Yang Z., Qin X., Yang K., Mao C. (2013). The efficacy and safety of alpha-1 blockers for benign prostatic hyperplasia: An overview of 15 systematic reviews. Curr. Med. Res. Opin..

[7] Gormley G.J., Stoner E., Bruskewitz R.C., Imperato-McGinley J., Walsh P.C., McConnell J.D., Andriole G.L., Geller J., Bracken B.R., Tenover J.S. (1992). The effect of finasteride in men with benign prostatic hyperplasia. N. Engl. J. Med..

[8] Lowe F.C., McConnell J.D., Hudson P.B., Romas N.A., Boake R., Lieber M., Elhilali M., Geller J., Imperto-McGinely J., Andriole G.L. (2003). Long-term 6-year experience with finasteride in patients with benign prostatic hyperplasia. Urology.

[9] Lecce L., Lam Y.T., Lindsay L.A., Yuen S.C., Simpson P.J., Handelsman D.J., Ng M.K. (2014). Aging impairs VEGF-mediated, androgen-dependent regulation of angiogenesis. Mol. Endocrinol..

[10] Uygur M., Gür E., Arik A., Altuǧ U., Erol D. (1998). Erectile dysfunction following treatments of benign prostatic hyperplasia: A prospective study. Andrologia.

[11] Chiba K., Yamaguchi K., Li F., Ando M., Fujisawa M. (2011). Finasteride-associated male infertility. Fertil. Steril..

[12] Sharma M., Chadha R., Dhingra N. (2017). Phytotherapeutic agents for benign prostatic hyperplasia: An overview. Mini Rev. Med. Chem..

[13] Kim S.-D., Lee B.-H., Sohn D.-W., Cho Y.-H., Lee S.-M., Kim J.-O., Kim S.W. (2008). The effect of herbal formulation KH-305 mainly consisted of *Rubus coreanus* on benign prostatic hyperplasia-induced rat. Korean J. Pharmacogn..

[14] Sun H., Li T.-J., Sun L.-N., Qiu Y., Huang B.-B., Yi B., Chen W.S. (2008). Inhibitory effect of traditional Chinese medicine Zi-Shen pill on benign prostatic hyperplasia in rats. J. Ethnopharmacol..

[15] Lee S.-H., Ahn Y.-M., Ahn S.-Y., Kim Y.-O., Lee B.-C. (2009). The antihyperplastic effect of oral *Curcuma longa* ingestion in a rat model of benign prostatic hyperplasia. Korean J. Intern. Med..

[16] Kim J.-S., Han Y.-H., Kim Y.-S. (2009). The effects of *Scutellaria baicalensis* on experimental rat model of benign prostatic hyperplasia. Korean J. Intern. Med..

[17] Shin I.S., Lee M.Y., Ha H.K., Seo C.S., Shin H.-K. (2012). Inhibitory effect of Yukmijihwang-tang, a traditional herbal formula against testosterone-induced benign prostatic hyperplasia in rats. BMC Complement. Altern. Med..

[18] Kim S.K., Seok H., Park H.J., Jeon H.S., Kang S.W., Lee B.-C., Yi J., Song S.Y., Lee S.H., Kim Y.O. (2015). Inhibitory effect of curcumin on testosterone induced benign prostatic hyperplasia rat model. BMC Complement. Altern. Med..

[19] Choi H.-M., Jung Y., Park J., Kim H.-L., Youn D.-H., Kang J., Jeong M.Y., Lee J.H., Yang W.M., Lee S.G. (2016). *Cinnamomi cortex* (*Cinnamomum verum*) suppresses testosterone-induced benign prostatic hyperplasia by regulating 5α-reductase. Sci. Rep..

[20] Busatta C., Vidal R., Popiolski A., Mossi A., Dariva C., Rodrigues M., Corazza F.C., Corazza M.L., Vladimir Oliveira J., Cansian R.L. (2008). Application of *Origanum majorana* L. essential oil as an antimicrobial agent in sausage. Food Microbiol..

[21] El-Meligy R.M., Awaad A.S., Soliman G.A., Kenawy S.A., Alqasoumi S.I. (2017). Prophylactic and curative anti-ulcerogenic activity and the possible mechanisms of action of some desert plants. Saudi Pharm. J..

[22] Soliman M.M., Nassan M.A., Ismail T.A. (2016). *Origanum majoranum* extract modulates gene expression, hepatic and renal changes in a rat model of type 2 diabetes. Iran. J. Pharm. Res. IJPR.

[23] Yazdanparast R., Shahriyary L. (2008). Comparative effects of *Artemisia dracunculus*, *Satureja hortensis* and *Origanum majorana* on inhibition of blood platelet adhesion, aggregation and secretion. Vasc. Pharmacol..

[24] Abdel-Massih R.M., Fares R., Bazzi S., El-Chami N., Baydoun E. (2010). The apoptotic and anti-proliferative activity of *Origanum majorana* extracts on human leukemic cell line. Leuk. Res..

[25] El-Ashmawy I.M., El-Nahas A.F., Salama O.M. (2005). Protective effect of volatile oil, alcoholic and aqueous extracts of *Origanum majorana* on lead acetate toxicity in mice. Basic Clin. Pharmacol. Toxicol..

[26] Seoudi D.M., Medhat A.M., Hewedi I.H., Osman S.A., Mohamed M.K., Arbid M.S. (2009). Evaluation of the anti-inflammatory, analgesic, and anti-pyretic effects of *Origanum majorana* ethanolic extract in experimental animals. J. Radiat. Res. Appl. Sci..

[27] Refaie A.A.E.-R., Ramadan A., Mossa A.-T.H. (2014). Oxidative damage and nephrotoxicity induced by prallethrin in rat and the protective effect of *Origanum majorana* essential oil. Asian Pac. J. Trop. Med..

[28] Ennaji H., Chahid D., Aitssi S., Badou A., Khlil N., Ibenmoussa S. (2020). Phytochemicals screening, cytotoxicity and antioxidant activity of the *Origanum majorana* growing in Casablanca, Morocco. Open Biol. Sci. J..

[29] Jaganathan S.K., Supriyanto E., Mandal M. (2013). Events associated with apoptotic effect of p-coumaric acid in HCT-15 colon cancer cells. World J. Gastroenterol. WJG.

[30] Chaves R.d.S.B., Martins R.L., Rodrigues A.B.L., de Menezes Rabelo É., Farias A.L.F., Araújo C.M.d.C.V., Almeida S.S. (2019). Larvicidal evaluation of the *Origanum majorana* L. essential oil against the larvae of the Aedes aegypti mosquito. bioRxiv.

[31] Nyati K.K., Prasad K.N., Rizwan A., Verma A., Paliwal V.K., Pradhan S. (2010). Lymphocyte transformation test detects a response to *Campylobacter jejuni* antigens in patients with Guillain-Barré syndrome. Med. Microbiol. Immunol..

[32] Charan J., Kantharia N. (2013). How to calculate sample size in animal studies?. J. Pharmacol. Pharmacother..

[33] Kim K.S., Yang H.Y., Chang S.C., Kim Y.M., Lee K.Y., Lee B.M., Kim H.S. (2018). Potential repositioning of GV1001 as a therapeutic agent for testosterone-induced benign prostatic hyperplasia. Int. J. Mol. Med..

[34] Choi B.R., Kim H.K., Soni K.K., Karna K.K., Lee S.W., So I., Park J.K. (2018). Additive effect of oral LDD175 to tamsulosin and finasteride in a benign prostate hyperplasia rat model. Drug Des. Devel. Ther..

[35] Abdel-Naim A.B., Neamatallah T., Eid B.G., Esmat A., Alamoudi A.J., El-Aziz A., Ashour O.M. (2018). 2-methoxyestradiol attenuates testosterone-induced benign prostate hyperplasia in rats through inhibition of HIF-1α/TGF-β/Smad2 axis. Oxid. Med. Cell. Longev..

[36] Soliman A.M., Desouky S., Marzouk M., Sayed A.A. (2016). *Origanum majorana* attenuates nephrotoxicity of cisplatin anticancer drug through ameliorating oxidative stress. Nutrients.

[37] Abdelsalam H.M., Diab A.A., Zahra M.H., Eldehamy S., Hendawy B.A. (2019). Therapeutic effects of *Origanum majorana* and propolis against nephrotoxicity induced by adenine. Biol. Eng. Med..

[38] Desouky S., Marzouk M., Soliman A.M., Sayed A.A. (2015). Modulatory effect of *Origanum majorana* extract against cisplatin-induced dyslipidemia in rats. Int. J. Curr. Res. Life Sci..

[39] Yang X., Yuan L., Xiong C., Yin C., Ruan J. (2014). *Abacopteris penangiana* exerts testosterone-induced benign prostatic hyperplasia protective effect through regulating inflammatory responses, reducing oxidative stress and anti-proliferative. J. Ethnopharmacol..

[40] Akbari F., Azadbakht M., Megha K., Dashti A., Vahedi L., Nejad A.B., Zahra M., Abdi Sarkami S., Sadati M. (2021). Evaluation of *Juniperus communis* L. seed extract on benign prostatic hyperplasia induced in male Wistar rats. Afr. J. Urol..

[41] An Y.J., Lee J.Y., Kim Y., Jun W., Lee Y.-H. (2020). Cranberry powder attenuates benign prostatic hyperplasia in rats. J. Med. Food.

[42] Khoubnasabjafari M., Ansarin K., Jouyban A. (2016). Critical review of malondialdehyde analysis in biological samples. Curr. Pharm. Anal..

[43] Kosova F., Temeltaş G., Arı Z., Lekili M. (2014). Possible relations between oxidative damage and apoptosis in benign prostate hyperplasia and prostate cancer patients. Tumor Biol..

[44] Erel O. (2004). A novel automated method to measure total antioxidant response against potent free radical reactions. Clin. Biochem..

[45] Aebi H. (1974). Catalase. Methods of Enzymatic Analysis.

[46] Sun Y., Oberley L.W., Li Y. (1988). A simple method for clinical assay of superoxide dismutase. Clin. Chem..

[47] Al-Trad B., Aljabali A., Al Zoubi M., Shehab M., Omari S. (2019). Effect of gold nanoparticles treatment on the testosterone-induced benign prostatic hyperplasia in rats. Int. J. Nanomed..

[48] Xu H., Chen Y., Chen Q., Xu H., Wang Y., Yu J., Zhou J., Wang X., Xu B. (2017). DNMT1 regulates IL-6-and TGF-β1-induced epithelial mesenchymal transition in prostate epithelial cells. Eur. J. Histochem. EJH.

[49] Bancroft J.D., Gamble M. (2008). Theory and Practice of Histological Techniques.

[50] Zhong W., Peng J., Wu D., Han Z., Bi X., Dai Q. (2008). Ki-67 and PCNA expression in prostate cancer and benign prostatic hyperplasia. Clin. Investig. Med..

[51] Shariat S.F., Ashfaq R., Roehrborn C.G., Slawin K.M., Lotan Y. (2005). Expression of survivin and apoptotic biomarkers in benign prostatic hyperplasia. J. Urol..

[52] Zheng C., Luo Y., Chen Y., Chen D., Shao C., Huang D., Zhu J., Mao X., Li L., Sun Z. (2020). Oral exposure of sulpiride promotes the proliferation of brown-Norway rat prostates. Exp. Ther. Med..

[53] Ramos-Vara J., Miller M. (2014). When tissue antigens and antibodies get along: Revisiting the technical aspects of immunohistochemistry—The red, brown, and blue technique. Vet. Pathol..

[54] Schindelin J., Rueden C.T., Hiner M.C., Eliceiri K.W. (2015). The ImageJ ecosystem: An open platform for biomedical image analysis. Mol. Reprod. Dev..

[55] Bankhead P., Loughrey M.B., Fernández J.A., Dombrowski Y., McArt D.G., Dunne P.D., McQuaid S., Gray R.T., Murray L.J., Coleman H.G. (2017). QuPath: Open source software for digital pathology image analysis. Sci. Rep..

[56] Fedchenko N., Reifenrath J. (2014). Different approaches for interpretation and reporting of immunohistochemistry analysis results in the bone tissue—A review. Diagn. Pathol..

[57] Iqbal B.M., Buch A. (2016). Hormone receptor (ER, PR, HER2/neu) status and proliferation index marker (Ki-67) in breast cancers: Their onco-pathological correlation, shortcomings and future trends. Med. J. Dr DY Patil Univ..

[58] Wu X., Gu Y., Li L. (2017). The anti-hyperplasia, anti-oxidative and anti-inflammatory properties of Qing Ye Dan and swertiamarin in testosterone-induced benign prostatic hyperplasia in rats. Toxicol. Lett..

[59] Jung Y., Park J., Kim H.-L., Youn D.-H., Kang J., Lim S., Jeong M.Y., Sethi G., Park S.J., Ahn K.S. (2017). Vanillic acid attenuates testosterone-induced benign prostatic hyperplasia in rats and inhibits proliferation of prostatic epithelial cells. Oncotarget.

[60] La Torre A., Giupponi G., Duffy D., Conca A., Cai T., Scardigli A. (2016). Sexual dysfunction related to drugs: A critical review. Part V: α-blocker and 5-ARI drugs. Pharmacopsychiatry.

[61] Garg G., Singh D., Saraf S., Saraf S. (2006). Management of benign prostate hyperplasia: An overview of α-adrenergic antagonist. Biol. Pharm. Bull..

[62] El-Ashmawy N.E., Khedr E.G., El-Bahrawy H.A., Helmy N.N. (2020). Modulatory effect of silymarin on apoptosis in testosterone-induced benign prostatic hyperplasia in rats. J. Clin. Oncol..

[63] Gheitasi I., Motaghi N., Sadeghi H., Sadeghi H., Moslemi Z., Eftekhari M., Shakerinasab N., Doustimotlagh A.H. (2021). Antioxidant and anti-inflammatory effects of *Origanum majorana* L. methanolic extract on bile duct ligation in male rats. Evid. Based Complement. Alternat. Med..

[64] Veličković D.T., Nikolova M.T., Ivancheva S.V., Stojanović J.B., Veljković V.B. (2007). Extraction of flavonoids from garden (*Salvia officinalis* L.) and glutinous (*Salvia glutinosa* L.) sage by ultrasonic and classical maceration. J. Serb. Chem. Soc..

[65] Ahmad M., Suhail N., Mansoor T., Banu N., Ahmad S. (2012). Evaluation of oxidative stress and DNA damage in benign prostatic hyperplasia patients and comparison with controls. Indian J. Clin. Biochem..

[66] Younis N.N., Elsherbiny N.M., Shaheen M.A., Elseweidy M.M. (2020). Modulation of NADPH oxidase and Nrf2/HO-1 pathway by vanillin in cisplatin-induced nephrotoxicity in rats. J. Pharm. Pharmacol..

[67] Eid B.G., Abdel-Naim A.B. (2020). Piceatannol attenuates testosterone-induced benign prostatic hyperplasia in rats by modulation of Nrf2/HO-1/NFκB axis. Front. Pharmacol..

[68] D’Amico R., Genovese T., Cordaro M., Siracusa R., Gugliandolo E., Peritore A.F., Interdonato L., Crupi R., Cuzzocrea S., Di Paola R. (2021). Palmitoylethanolamide/baicalein regulates the androgen receptor signaling and NF-κB/Nrf2 pathways in benign prostatic hyperplasia. Antioxidants.

[69] Chen Y., Xu H., Liu C., Gu M., Chen Q., Zhan M., Wang Z. (2021). Therapeutic effects of 25-hydroxyvitamin D on the pathological process of benign prostatic hyperplasia: An in vitro evidence. Dis. Markers.

[70] Cadet J., Douki T., Gasparutto D., Ravanat J.-L. (2003). Oxidative damage to DNA: Formation, measurement and biochemical features. Mutat. Res. Fundam. Mol. Mech. Mutagen..

[71] Wang J., Yi J. (2008). Cancer cell killing via ROS: To increase or decrease, that is the question. Cancer Biol. Ther..

[72] Trachootham D., Zhou Y., Zhang H., Demizu Y., Chen Z., Pelicano H., Chiao P.J., Achanta G., Arlinghaus R.B., Liu J. (2006). Selective killing of oncogenically transformed cells through a ROS-mediated mechanism by β-phenylethyl isothiocyanate. Cancer Cell..

[73] Stadtman E.R., Berlett B.S. (1998). Reactive oxygen-mediated protein oxidation in aging and disease. Drug Metab. Rev..

[74] Na H.K., Oliynyk S. (2011). Effects of physical activity on cancer prevention. Ann. N. Y. Acad. Sci..

[75] Al Dhaheri Y., Eid A., AbuQamar S., Attoub S., Khasawneh M., Aiche G., Hisaindee S., Iratni R. (2013). Mitotic arrest and apoptosis in breast cancer cells induced by *Origanum majorana* extract: Upregulation of TNF-α and downregulation of survivin and mutant p53. PLoS ONE.

[76] Rao S., Timsina B., Nadumane V.K. (2014). Evaluation of the anticancer potentials of *Origanum marjorana* on fibrosarcoma (HT-1080) cell line. Asian Pac. J. Trop. Dis..

[77] Hajlaoui H., Mighri H., Aouni M., Gharsallah N., Kadri A. (2016). Chemical composition and in vitro evaluation of antioxidant, antimicrobial, cytotoxicity and anti-acetylcholinesterase properties of Tunisian *Origanum majorana* L. essential oil. Microb. Pathog..

[78] García-Risco M.R., Mouhid L., Salas-Pérez L., López-Padilla A., Santoyo S., Jaime L., Ramírez de Molina A., Reglero G., Fornari T. (2017). Biological activities of Asteraceae (*Achillea millefolium* and *Calendula officinalis*) and Lamiaceae (*Melissa officinalis* and *Origanum majorana*) plant extracts. Plant Foods Hum. Nutr..

[79] Makrane H., El Messaoudi M., Melhaoui A., El Mzibri M., Benbacer L., Aziz M. (2018). Cytotoxicity of the aqueous extract and organic fractions from *Origanum majorana* on human breast cell line MDA-MB-231 and human colon cell line HT-29. Adv. Pharmacol. Sci..

[80] Benhalilou N., Alsamri H., Alneyadi A., Athamneh K., Alrashedi A., Altamimi N., Al Dhaheri Y., Eid A.H., Iratni R. (2019). *Origanum majorana* ethanolic extract promotes colorectal cancer cell death by triggering abortive autophagy and activation of the extrinsic apoptotic pathway. Front. Oncol..

[81] Pakdemirli A., Karaca C., Sever T., Daşkin E., Leblebici A., Yiğitbaşi T., Başbinar Y. (2020). Carvacrol alters soluble factors in HCT-116 and HT-29 cell lines. Turk. J. Med. Sci..

[82] Elbe H., Yigitturk G., Cavusoglu T., Baygar T., Ozgul Onal M., Ozturk F. (2020). Comparison of ultrastructural changes and the anticarcinogenic effects of thymol and carvacrol on ovarian cancer cells: Which is more effective?. Ultrastruct. Pathol..

[83] Bouyahya A., Belmehdi O., Benjouad A., El Hassani R.A., Amzazi S., Dakka N., Bakri Y. (2020). Pharmacological properties and mechanism insights of Moroccan anticancer medicinal plants: What are the next steps?. Ind. Crops Prod..

[84] Marti A., Jaggi R., Vallan C., Ritter P.M., Baltzer A., Srinivasan A., Dharmarajan A.M., Friis R.R. (1999). Physiological apoptosis in hormone-dependent tissues: Involvement of caspases. Cell Death Differ..

[85] Omezzine A., Mauduit C., Tabone E., Nabli N., Bouslama A., Benahmed M. (2003). Caspase-3 and-6 expression and activation are targeted by hormone action in the rat ventral prostate during the apoptotic cell death process. Biol. Reprod..

[86] Culig Z., Hobisch A., Cronauer M.V., Radmayr C., Hittmair A., Zhang J., Thurnher M., Bartsch G., Klocker H. (1996). Regulation of prostatic growth and function by peptide growth factors. Prostate.

[87] Tuxhorn J.A., Ayala G.E., Smith M.J., Smith V.C., Dang T.D., Rowley D.R. (2002). Reactive stroma in human prostate cancer: Induction of myofibroblast phenotype and extracellular matrix remodeling. Clin. Cancer Res..

[88] Rick F.G., Schally A.V., Block N.L., Halmos G., Perez R., Fernandez J.B., Vidaurre I., Szalontay L. (2011). LHRH antagonist Cetrorelix reduces prostate size and gene expression of proinflammatory cytokines and growth factors in a rat model of benign prostatic hyperplasia. Prostate.

[89] Lai K.-P., Huang C.-K., Fang L.-Y., Izumi K., Lo C.-W., Wood R., Kindblom J., Yeh S., Chang C. (2013). Targeting stromal androgen receptor suppresses prolactin-driven benign prostatic hyperplasia (BPH). Mol. Endocrinol..

[90] Said E., Zaitone S.A., Eldosoky M., Elsherbiny N.M. (2018). Nifuroxazide, a STAT3 inhibitor, mitigates inflammatory burden and protects against diabetes-induced nephropathy in rats. Chem. Biol. Interact..

[91] Cardenas H., Vieth E., Lee J., Segar M., Liu Y., Nephew K.P., Matei D. (2014). TGF-β induces global changes in DNA methylation during the epithelial-to-mesenchymal transition in ovarian cancer cells. Epigenetics.

