# Supplementary material for: Origanum majorana L. Extract Attenuated Benign Prostatic Hyperplasia in Rat Model: Effect on Oxidative Stress, Apoptosis, and Proliferation
Source: Antioxidants (Basel). 2022 Jun 11;11(6):1149. doi: 10.3390/antiox11061149 (PMC9219805; doi:10.3390/antiox11061149)
Supplement: Supplementary file 1 [file antioxidants-11-01149-s001.zip › antioxidants-1689419-supplementary.pdf]

Supplementary materials

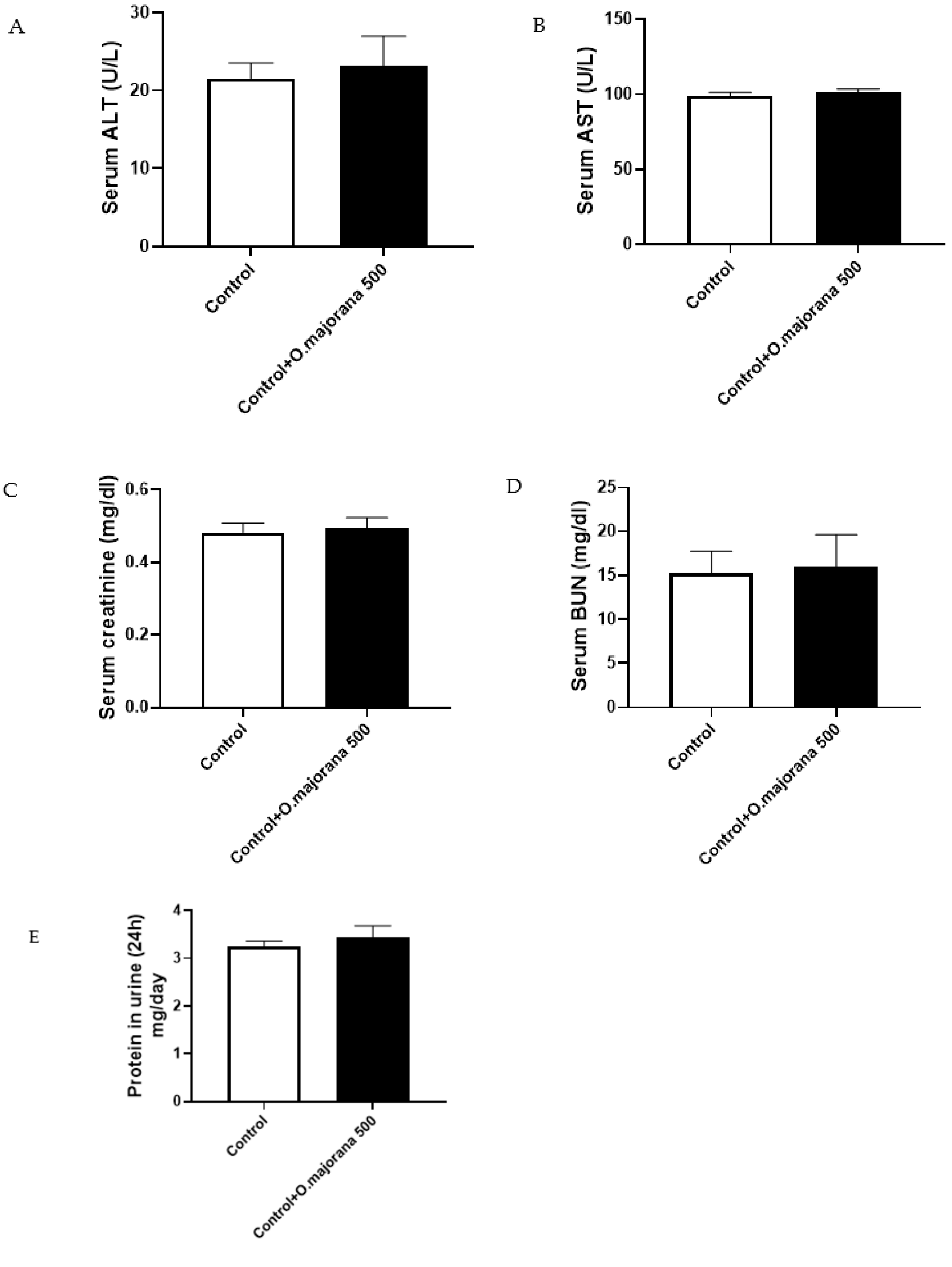

Figure S1. Effect of *O. majorana (*500 mg/kg/day) on (**A**) serum alanine aminotransferase (ALT), (**B**) serum aspartate aminotransferase (AST), (**C**) serum creatinine, (**D**) blood urea nitrogen (BUN), (**E**) protein in urine when administrated to normal control group.

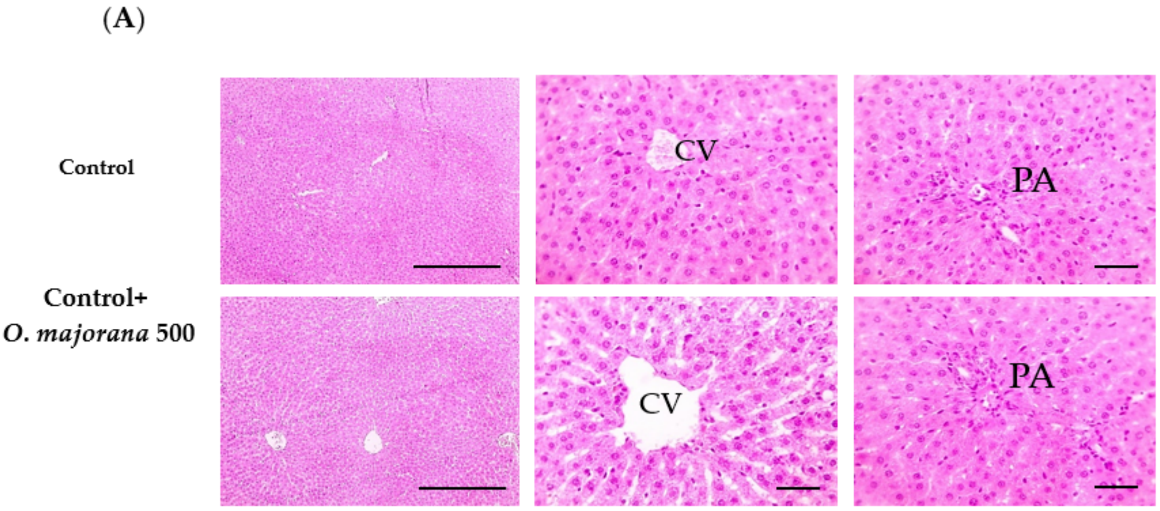

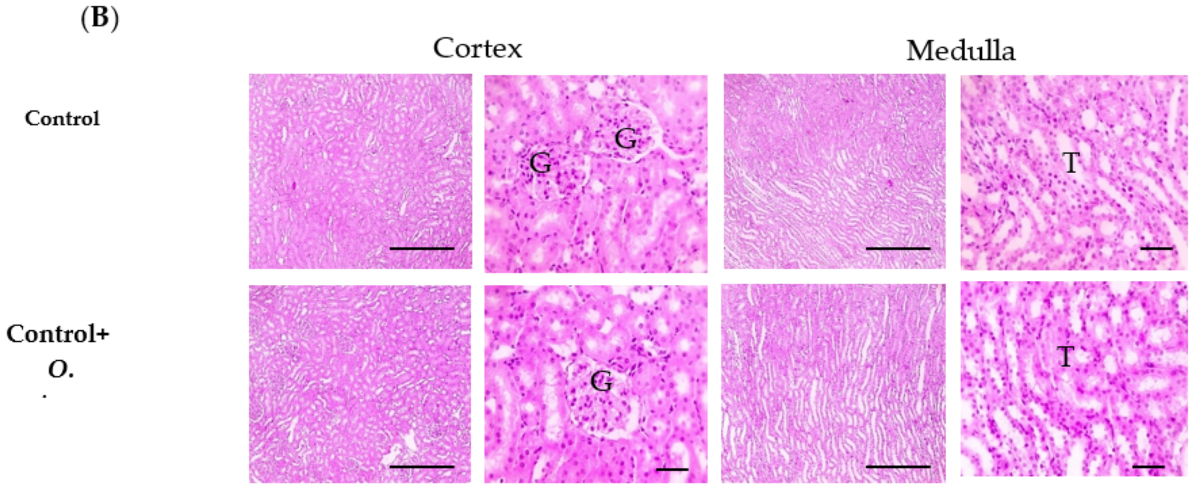

Figure S2. (**A**) Microscopic pictures of H&E-stained hepatic sections showing normal hepatocytes, central vein (CV) and portal areas (PA) in control group, and control treated with *O. majorana* 500mg/kg/day . Low magnification X: 100 bar 100 and high magnification X: 400 bar 50. (**B**) Microscopic pictures of H&E-stained renal sections showing normal cortex including glomeruli (G) and tubules (T) and medulla in control group and control treated with *O. majorana* 500mg/kg/day. Low magnification X: 100 bar 100 and high magnification X: 400 bar 50.
